# Shared genetic architecture of psychoactive substance use and pan-cancer: insights from a large‑scale genome‑wide cross‑trait analysis

**DOI:** 10.1186/s12916-026-04677-3

**Published:** 2026-02-05

**Authors:** Jiahang Song, Pengzhu Li, Martin Canis, Kristian Unger, Nikolaus Alexander Haas, Olivier Gires

**Affiliations:** 1 Department of Otorhinolaryngology, LMU University Hospital, LMU Munich, Munich, Germany; 2 Division of Pediatric Cardiology and Intensive Care, University Hospital, LMU Munich, Munich, Germany; 3 Department of Radiation Oncology, University Hospital, LMU Munich, Munich, Germany

**Keywords:** GWAS, Psychoactive substance use (PSU), Cancer, PSU-cancer trait pairs, CHRNA2, HRH3, PTK6

## Abstract

**Background:**

Psychoactive substance use (PSU) and cancer are frequently observed comorbidities that have reciprocal influences and shared behavioral traits of the affected patients. While, e.g., nicotine and alcohol are major carcinogens in the etiology of lung and head and neck cancers, little is known about a shared overarching genetic architecture of PSU and cancer that may predispose individuals to both illnesses.

**Methods:**

Large-scale genome-wide association study (GWAS) summary data revealed shared genetic architecture between cancer and PSU, including alcohol use dependence (AlcUD) and nicotine use dependence (NicUD). Genetic correlations between PSU and cancer were assessed by linkage disequilibrium score regression (LDSC) and high-definition likelihood (HDL). Mendelian randomization (MR) analysis was additionally employed to explore causal associations between PSU and cancer. Moreover, phenome-wide association study (PheWAS) and drug target analysis were utilized to evaluate the safety and therapeutic value of pleiotropic hub genes.

**Results:**

GWAS-based cross-referencing of PSU and cancer identified 34 shared trait pairs with significant genetic correlations and a total of 97 pleiotropic genomic risk loci. Affected loci mapped to genes expressed in the brain cerebellum (*n* = 109) and included cross-trait pleiotropic hub genes (*n* = 21). MR analysis further identified causal effects of AlcUD and NicUD on cancer risk. After exclusion of genes at high risk of side effects upon inhibition in a PheWAS, cholinergic receptor nicotinic alpha 2 (CHRNA2), histamine receptor H3 (HRH3), and protein tyrosine kinase 6 (PTK6) were identified as potentially druggable targets.

**Conclusions:**

In summary, we identified a shared genetic architecture comprising pleiotropic cerebellar hub genes linking PSU-cancer trait pairs and described potential interventional drugs.

**Supplementary Information:**

The online version contains supplementary material available at 10.1186/s12916-026-04677-3.

## Background

Substance use disorders (SUD) and psychoactive substance use (PSU) are very serious conditions that are associated with high relapse rates and morbidity. SUD/PSU have an additional health impact as they potentiate the development of illnesses including chronic pain, cardiovascular diseases, mental health disorders, and cancer [1–6]. In the following, we will refer to PSU as a converging term encompassing substance-related phenotypes such as use disorders or dependence and non-pathological use or consumption traits. The term cancer covers over 200 malignant diseases with pronounced inter- and intratumor molecular heterogeneity and variations in treatment response due to diverse underlying pathobiological, molecular mechanisms, and co-morbidities. PSU and tumorigenesis are both driven to varying degrees by genetic, epigenetic, environmental, and behavioral factors, and together cause major health and economic problems worldwide [1, 4, 7, 8].

Alcohol and nicotine dependence constitute major forms of PSU disorders with high global prevalence [9, 10]. Chronic and heavy consumption of alcohol and tobacco has been shown to greatly contribute to cancer development and progression [11–13]. For example, nicotine and alcohol abuse are major risk factors for head and neck squamous cell carcinoma [14–16]. In recent years, accumulating evidence has suggested shared genetic associations between alcohol dependence and cancers of the respiratory and digestive systems, as well as between nicotine dependence and lung cancer [17]. Cannabis is the most widely smoked substance after tobacco, and its prevalence continues to rise with the expansion of legal markets [18]. The increasing legalization and medical use of cannabis have prompted extensive research into its potential therapeutic effects on cancer-related symptoms such as pain, nausea, and loss of appetite, making it a possible adjunct therapy in cancer management [19, 20]. However, the association between cannabis use and cancer risk remains controversial. A large-scale cohort study reported that individuals with cannabis use disorder (CanUD) had more than a threefold increased risk of developing oral cancer compared with those without CanUD [21]. In contrast, some researchers have suggested a potential antitumor effect of cannabinoids, with evidence showing a significantly reduced risk of prostate cancer among individuals aged 50–64 who were current or former cannabis smokers [22]. Opioids, including prescription analgesics such as morphine, are widely used for pain management in cancer patients [23]. However, their extensive misuse and addictive potential have contributed to a global opioid crisis. Beyond their analgesic properties, opioids may regulate cancer progression through interactions with opioid receptors, which are closely associated with tumor growth and metastasis [24]. An observational study found that chronic opioid use among patients with persistent pain was associated with an increased risk of several cancers, including breast cancer [25]. Conversely, other studies have reported anti-tumor effects of opioids in leukemia [26]. Tea and coffee are among the most consumed beverages worldwide. Owing to their abundance of bioactive compounds such as catechins, tea polyphenols, caffeine, and various antioxidants [27, 28], researchers have shown great interest in their potential associations with cancer risk [29, 30]. A meta-analysis has suggested an inverse association between tea consumption and multiple types of cancer [31–33]. Similarly, some observational studies have suggested that coffee intake may reduce the risk of several cancers, including prostate, renal cell, and breast cancers [34–37], whereas other studies have reported conflicting findings [29, 38, 39]. However, traditional observational studies are inherently limited by residual confounding, selection bias, and measurement errors, which may obscure true relationships between PSU and cancer risk. Growing attention has been devoted to identifying common genetic foundations across diverse complex traits [40–42]. A previous study has revealed that SUD shares a common genetic structure with chronic pain [43]. Similarly, alcohol use disorder and body mass index exhibit genetic pleiotropy and shared neural associations [44]. Moreover, mood disorders and substance misuse have been shown to possess overlapping genetic architecture [45]. However, there is currently no systematic study exploring a potentially shared genetic architecture between PSU and cancer. Recent advances in genetic research have provided new perspectives on PSU and cancer, as genome-wide association studies (GWASs) have identified multiple genetic variants associated with each trait [46–51]. An underlying genetic architecture pre-dating sequential mutagenesis entailed by carcinogenic agents may account for an additional layer of heterogeneity [52]. The identification of such predispositions towards linked diseases rooted in population-wide genetic variations may help to better understand the course of life-threatening diseases. Therefore, we have conducted a large-scale cross-trait GWAS to identify common genetic architectures of PSU-cancer pairs in humans. We demonstrated the existence of shared loci and hub genes affected across trait pairs. MR methods further uncovered causal effects of PSU (AlcUD and NicUD) on cancer risk. These hub genes are primarily expressed in the brain cerebellum and encode druggable target candidates. Thus, we report on genetic architectures linking substance use and cancer development and identified common, actionable genes affected in various PSU-cancer trait pairs.

## Methods

### GWAS summary data source

(i) GWAS summary statistics for alcohol use dependence (AlcUD) [46] and cannabis use disorder (CanUD) [48] were obtained from the Psychiatric Genomics Consortium (PGC). Summary statistics for nicotine use dependence (NicUD) [53] were collected from the GWAS and Sequencing Consortium of Alcohol and Nicotine Use (GSCAN). Summary statistics for aspirin use (AspU) [54], opioid use (OpiU) [54], coffee use (CofU), and tea use (TeaU) were obtained from the UK Biobank [55]. Summary statistics for hypnotics use (HypU) were collected from the FinnGen R11 database [56]. In this study, PSU is used as an umbrella term encompassing a spectrum of substance-related phenotypes, ranging from use disorders or dependence (e.g., AlcUD, NicUD, CanUD) to non-pathological use or consumption traits (e.g., AspU, OpiU, HypU, CofU, and TeaU). Notably, not all PSU traits represent aberrant or disordered use.

(ii) GWAS summary statistics for 20 types of cancer were retrieved from public large-scale GWAS or GWAS meta-analyses: glioma (GLIOMA) [57], low-grade gliomas (LGG) [57], glioblastoma (GBM) [57], esophageal adenocarcinoma (EAC) [58, 59], lung cancer (LC) [50], lung adenocarcinoma (LUAD) [50], lung squamous cell carcinoma (LUSC) [50], small cell lung cancer (SCLC) [50], breast cancer (BRCA) [49], breast cancer with positive estrogen receptor (BRCA ER^+^) [49], breast cancer with negative estrogen receptor (BRCA ER^−^) [49], colon cancer (COAD) [51], rectum cancer (READ) [51], bladder cancer (BLCA) [51], renal cell carcinoma (RCC) [51], cervical cancer (CESC) [51], uterine corpus endometrial cancer (UCEC) [51], malignant melanoma (MELA) [51], prostate cancer (PRAD) [60, 61], and ovarian cancer (OCAC) [62]. General information on these GWAS studies is provided in Additional File 2: Table S1.

### Quality control for SNPs

All GWAS summary statistics were harmonized to the GRCh37 (hg19) reference genome prior to analysis. SNP-level quality control was performed following standard GWAS practices to ensure data consistency across traits. Filtering criteria are as follows: (i) exclusion of non-bipartite allele SNPs and SNPs with strand-ambiguous alleles; (ii) exclusion of SNPs without rs tags; (iii) deletion of duplicated SNPs, SNPs excluded in the 1000 Genomes Project, or with mismatched alleles; (iv) exclusion of SNPs within the major histocompatibility complex region at chr6: 28.5–33.5 Mb from LDSC analysis; (v) retention of SNPs with minor allele frequency (MAF) > 0.01.

### Genome-wide genetic correlations analysis

Global genetic correlations (rg) between PSU phenotype and cancer were assessed by linkage disequilibrium score regression (LDSC) [63] and high-definition likelihood (HDL) [64]. LDSC quantifies the genome-wide covariance in SNP effect sizes between two traits, thereby capturing polygenic genetic correlation across the entire genome. The 1000 Genomes Project Phase 3 European super-population served as the linkage disequilibrium (LD) reference panel, and SNPs were filtered to the HapMap3 set recommended by the LDSC developers. The baselineLD_v2.2 model was applied to control for potential confounding due to functional annotation and LD structure. The LD scores used in LDSC were calculated from common SNPs (minor allele frequency > 0.01) in the 1000 Genomes reference panel. Standard errors were estimated using the block jackknife method, and the LDSC intercept was used to assess potential sample overlap or residual population stratification between datasets. All GWAS summary statistics were preprocessed with the “munge_sumstats.py” script to harmonize alleles, align effect directions, and remove ambiguous or mismatched SNPs. This procedure ensured that all datasets were aligned to a consistent genomic build (GRCh37/hg19) and reference allele orientation before downstream analyses. The reference panel including 1,029,876 quality-controlled HapMap3 SNPs was used for HDL analysis.

### Identification of pleiotropic loci and genes

Pleiotropic analysis under composite null hypothesis (PLACO) analysis served to systematically identify genetic associations between PSU and cancer at SNP level. PLACO is a statistical approach specifically designed to detect pleiotropy, enabling the identification of shared genetic variants across multiple phenotypes [65]. The composite null hypothesis in PLACO assumes that a given SNP is associated with at most one trait, that is, either associated with trait 1 only, trait 2 only, or neither trait. The alternative hypothesis corresponds to a true bivariate association, where the SNP is simultaneously associated with both traits. PLACO tests this composite null using a product-based test statistic that combines *Z*-scores from the two GWASs, providing greater sensitivity for detecting shared associations while maintaining strict type I error control. We considered SNPs that reached genome-wide significance (*P* < 5 × 10^−8^) as pleiotropic variants, indicating that these SNPs show strong genetic associations across multiple phenotypes. Identification of pleiotropic variants is essential for uncovering the shared genetic basis of PSU and cancer. Functional Mapping and Annotation (FUMA) was used to map risk variants to specific genomic regions. FUMA localizes SNPs to specific genomic regions (i.e., risk loci), providing deeper insights into potential functions of these variants [66]. Lead SNPs were used to map nearby genes within each locus and generalized gene-set analysis of GWAS data served to identify biological functions of pleiotropic loci. Locus definition was conducted using the 1000 Genomes Project Phase 3 European (1000G Phase3 EUR) reference panel with the following parameter settings: maximum *P*-value for lead SNPs = 5 × 10⁻⁸, maximum P-value cutoff for candidate SNPs = 0.05, r^2^ threshold to define independent significant SNPs = 0.6, secondary *r*^2^ threshold to define lead SNPs = 0.1, minimum minor allele frequency (MAF) = 0, and maximum distance between LD blocks to merge into one locus = 250 kb.

Multi-marker analysis of genomic annotation (MAGMA) was conducted to determine pleiotropic genes via the implementation of linkage disequilibrium between markers and to detect multi-marker effects [67]. SNPs were mapped to genes according to the GENCODE v19 (GRCh37/hg19) annotation with a ± 50 kb window around gene boundaries, and the same 1000 Genomes Phase 3 European reference panel was used to account for linkage disequilibrium among markers. MAGMA first aggregates SNP-level associations into gene-level statistics and then evaluates enrichment across predefined biological pathways. 10,678 gene sets including curated gene sets (c2.all) and gene ontology (GO) terms (c5.bp, c5.cc, and c5.mf) from Molecular Signatures Database (MSigDB) were tested [68]. Bonferroni correction was used for all tested gene sets to avoid false positives (*P* < 0.05/10,678 = 4.68 × 10^−6^).

### Colocalization analysis

R package coloc [69] served to determine co-localizations of association signals for PSU and cancer. For each of the shared SNPs between trait pairs, variants in the range of 500 Kb of the index SNP were extracted and the probability that the two traits share one common causal variant (H4) was calculated. Colocalization was considered for loci with a probability greater than 0.7. The posterior probability (PP) of multiple traits sharing the same SNP was estimated using a Bayesian divisive clustering algorithm implemented by the HyPrColoc package in R [70].

### Tissue-enrichment analysis

Genome-wide tissue-specific enrichment analysis (GWTSEA) was conducted based on 30 general GTEx tissues [69] for MAGMA-derived genome-wide pleiotropic loci [68]. Considering that the brain was significantly enriched in all trait pairs, we further perform GWTSEA based on 13 GTEx brain tissues at the GTEx website (https://www.gtexportal.org/home/tissue/).

### Single-cell level specificity

Single-cell disease relevance score (scDRS) relates scRNAseq data to polygenic disease risk at single-cell resolution, independently of the annotated cell type [71]. We applied scDRS (version v1.0.2) to assess cell-level disease associations (https://github.com/martinjzhang/scDRS). The scDRS framework estimates disease relevance at the single-cell level by integrating GWAS-based polygenic risk signals with single-cell transcriptomic data. It performs a gene set enrichment analysis using a gene set whose members are weighted by their association strength with the trait of interest, derived from an external method. In this study, the gene-level *P*-value output from MAGMA served as the input to the scdrs munge-gs function. For each cell, scDRS computes a raw disease relevance score based on the expression of trait-associated genes and then generates multiple Monte Carlo (MC) control scores from matched random gene sets with similar expression characteristics. A unified MC test is subsequently used to obtain an empirical P-value for each cell, identifying cells whose transcriptomic profiles show significant enrichment for disease-associated genes. The method further performs downstream analyses to identify (i) significant pre-annotated cell states based on aggregated group *Z* scores and (ii) individual genes whose expression levels correlate with the inferred disease relevance scores. All analyses were conducted using default parameter settings as recommended in the official repository.

### Gene-based association analysis

We used MAGMA, transcriptome-wide association study (TWAS)-fusion, and summary-based Mendelian randomization (SMR) to identify common genes shared across trait pairs. Genome-wide gene-based association studies (GWGAS) were conducted using MAGMA [68], integrating SNP-based *P*-values from GWAS, including 19,427 protein-coding genes (NCBI 37.3 gene annotations). Gene association tests considered linkage disequilibrium (LD) of SNPs, ensuring accurate assessment of gene-level associations. Genes exhibiting a *P*-value below the threshold of 0.05 were selected for subsequent analysis.

TWAS for was performed using FUSION (http://gusevlab.org/projects/fusion/) [72]. Reference weights for gene expression in the target tissue were calculated with multiple prediction models in FUSION. SNPs within 1 Mb of a given gene were selected from GWAS summary statistics. The imputation *Z* score for the *cis* genetic effect on each trait was calculated using the formula ZTWAS = W’Z/(W’SW)1/2. *Z* represents GWAS summary *Z*-scores, *W* represents weights, and *S* the SNP-correlation covariance matrix. Genes with *P*-values < 0.05 after 5000 permutations were selected for further analysis.

SMR [73] associated GWAS summary-level data with expression quantitative trait loci (eQTL) studies to identify pleiotropic genes associated with complex traits. It employs SMR and heterogeneity in dependent instruments (HEIDI) methods to test pleiotropic associations between gene expression levels and complex traits of interest using summary-level data from GWAS and eQTL studies. This approach tests whether the magnitude of SNP effects on phenotypes is mediated by gene expression. Genes with a permutated *P*-value < 0.05 were selected for subsequent analysis.

### Causal association analysis

Two-sample Mendelian randomization (MR) analysis was conducted to determine potential causal effects between PSU and cancer traits. Linkage disequilibrium (*r*^2^) clumping was employed in PLINK 1.9 to obtain independent significance SNPs (*P* < 5 × 10⁻⁸) for all exposure traits, using an *r*^2^ threshold of 0.001 within a 10,000 kb window [74] based on the 1000 Genomes Project Phase 3 reference panel. The strength of the instrumental variables (IVs) was evaluated using the proportion of variance explained (PVE) and the F statistic (*F* > 10) [75]. Causal effects between each trait pair were assessed using four MR methods: inverse variance weighted (IVW) [76], MR Egger [77], RAPS [78], and CAUSE [79]. Cochran’s *Q* statistics were applied to detect the effect size heterogeneity across the IVs [80]. A false discovery rate (FDR) of 5% was used as the threshold.

### Mediation analysis

Two-step MR based mediation analysis was employed to disclose whether PSU mediates the causal pathway from hub gene to cancer outcome. The overall effect can be divided into an indirect effect (through mediators) and a direct effect (without mediators) effect [81]. The total effect of PSU on cancer was divided into 1) direct effects of PSU on cancer and 2) indirect effects mediated by PSU through the mediator. The proportion of mediating effect was obtained by dividing the indirect effect by the total effect, with 95% confidence intervals determined via the delta method.

### Phenome-wide association study (PheWAS)

A PheWAS with the ExPheWas online tool (https://exphewas.statgen.org/) served to identify horizontal pleiotropy of potential drug targets and possible side effects [82]. Multiple corrections were performed, and a threshold of 5 × 10^−8^ was set as the default in the ExPheWas Portal to account for the potential for false positives.

### Candidate drug prediction

Assessing protein-drug interactions was conducted using the Drug-Gene Interaction database (DGIdb, https://www.dgidb.org/) to screen potential drugs [83]. DGIdb is a database of drug-gene interactions that provides information on known or potential associations between genes and drugs. It is currently updated to version 5.0. DGIdb contains over 14,000 drug-gene interactions, involving 2600 genes and 6300 targeting drugs. Additionally, it includes 6,700 additional genes that are likely to become drug targets in the future.

### Molecular docking analysis

Molecular docking was performed to evaluate the interactions between hub proteins and their corresponding candidate drugs predicted by the DGIdb database. Docking simulations were conducted using AutoDock Vina (version v1.1.2) [84], and protein structures were obtained from the Protein Data Bank (PDB) or AlphaFold when experimental structures were unavailable. Proteins were prepared by removing water molecules, adding polar hydrogens, and assigning Gasteiger charges using AutoDockTools. Ligand structures were retrieved from DGIdb databases and energy-minimized using the MMFF94 force field. A docking grid was defined to cover the predicted binding pocket of each protein. For each protein–drug pair, nine docking poses were generated, and the pose with the lowest binding energy was selected for further interpretation. Docking affinity scores (kcal/mol) and interaction profiles were analyzed to assess druggability, and top-ranked complexes were visualized using PyMOL (version v 3.1.3).

### Software and packages

Statistical analysis was performed in R (version v4.1.1). LDSC analysis was conducted with “LDSC” software (version v1.0.1) [63]. PLACO was performed with the “PLACO” package [65]. Bayesian colocalization analysis was performed with the “coloc” package (version v5.2.1) [85]. Function analysis was performed with the FUMA web tool [66]. MAGMA gene and gene-set analysis were performed with the MAGMA software [67]. Two-sample MR analysis was conducted with “MendelianRandomization” (version v0.10.0) [86].

## Results

### Genetic correlations between PSU phenotypes and cancer

**Fig. 1:**
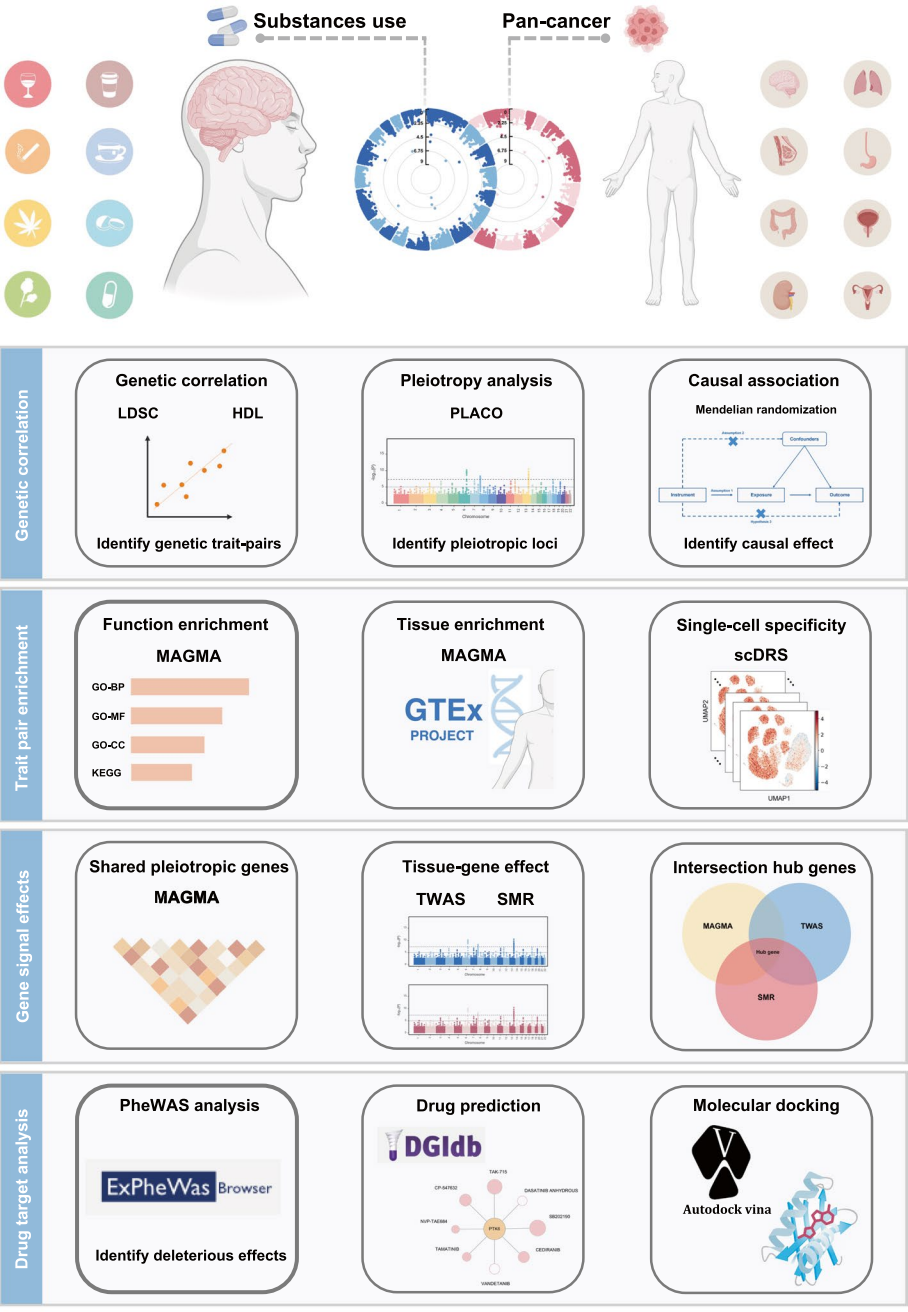
Schematic representation of the overall study design. Shared SNPs associated with psychoactive substance use (PSU) and pan-cancer entities were identified via LDSC and HDL. Pleiotropy of identified SNPs/loci was conducted with PLACO. Causal association between PSU and cancer was detected by MR analysis. Functional and expression analysis of SNPs/loci was addressed with MAGMA, data from the GTEx project, and scRNAseq datasets of cerebellum regions. Shared hub genes were analyzed for potential effects on tissue (PheWAS) and for the availability of clinical drugs

The present study interrogated shared genetic architectures between PSU and tumor entities in GWAS summary-level data. First, we evaluated the global genetic correlations between PSU and cancer trait pairs and identified shared pleiotropic loci. Furthermore, the causal relationship between PSU and cancer was detected by MR analyses. The shared biological mechanism of these trait pairs was then characterized by enriched pathways, relevant organs or tissues, and specific cell subtypes. Shared pleiotropic genes were subsequently obtained through TWAS and SMR analyses. Finally, we performed drug-target prediction based on the identified pleiotropic genes and constructed a regulatory network to highlight potential therapeutic targets (Fig. 1).

**Fig. 2:**
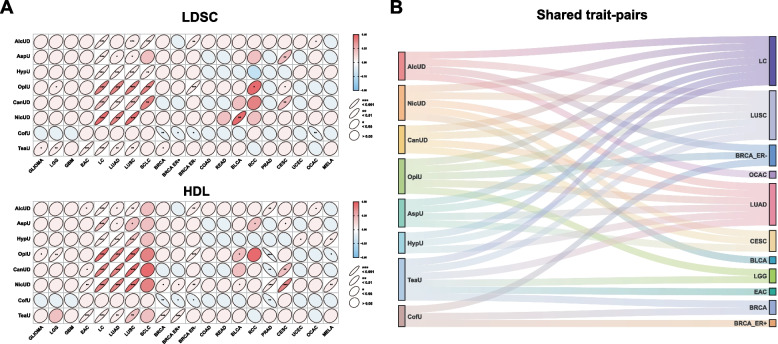
Global genetic correlations between psychoactive substance use and pan-cancer. **A** Genetic correlation analysis between PSU and cancer entities using LDSC and HDL. Correlation levels are indicated by the shape of the symbol, correlation strength by color-coding, and correlation directionality by left (negative) or right tilt (positive). **B** Shared trait pairs between LDSC and HDL are depicted in a Sankey diagram including PSU and the indicated cancer entities. Abbreviations: alcohol use dependence (AlcUD), nicotine use dependence (NicUD), cannabis use disorder (CanUD), opioid use (OpiU), aspirin use (AspU), hypnotics use (HypU), tea use (TeaU), and coffee use (CofU)

We first estimated the SNP-based heritability (h_2_^SNP^) for PSU and cancer traits. Phenotypes, sample size, ratio of case-to-controls, source, and PubMed ID are summarized in Additional file 2: Table S1. This cross-trait GWAS aggregated 6,145,925 samples with 598,931 cases, and case proportions of 0.49–56.43%. The results indicated significant heritability for all traits and reflected the polygenic character of each trait. We employed both LDSC and HDL to assess global genetic correlations (*r*_*g*_) for each trait pair composed of one PSU and one cancer subtype individually (Fig. 2A). A total of eight PSU traits including AlcUD, AspU, HypU, OpiU, CanUD, NicUD, CofU, and TeaU were cross-referenced with *n* = 20 tumor types, resulting in 34 shared trait pairs with significant genetic correlations identified with LDSC and HDL (Fig. 2B). PSU showed a positive genetic correlation with *n* = 11/20 cancer types. Notably, all PSU traits except CofU showed significant positive genetic correlations with LC-related traits. All BC traits exhibited remarkable negative genetic correlations with CofU (Additional file 2: Table S2 and Fig. 2B).

### Shared loci and genes between PSU phenotypes and cancer

**Fig. 3:**
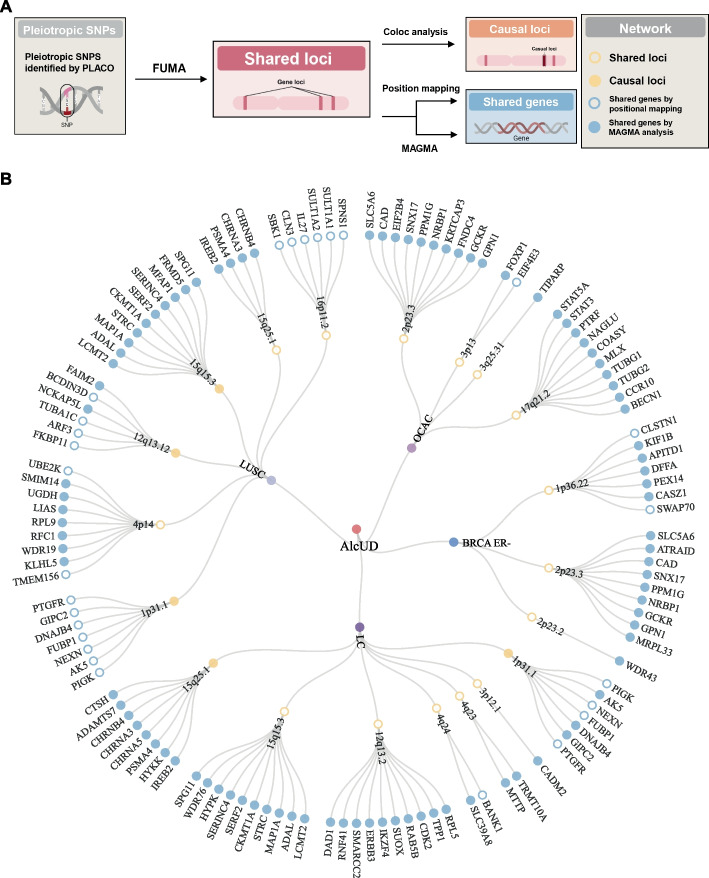
Pleiotropic associations across alcohol use and pan-cancer. **A** Schematic representation of the study workflow designed to identify shared and causal loci/genes of trait pairs. **B** Circular network showing the pleiotropic landscape between alcohol use dependence (AlcUD) and estrogen receptor-negative breast cancer (BRCA-ER −), lung cancer (LC), lung squamous cell carcinoma (LUSC), and ovarian cancer (OCAC). The circular network includes SNP-affected chromosomal loci (yellow circles) and genes (blue circles) and distinguishes shared and causal loci (open/closed circles), and shared genes identified by positional mapping and by MAGMA analysis (open/closed circles)

PLACO was employed to explore potential pleiotropic loci for each trait pair identified with LDSC and HDL. Subsequently, we conducted a functional mapping and annotation of genetic associations of PLACO results using FUMA [65] (Fig. 3A). A total of 97 pleiotropic genomic risk loci significantly correlated with PSU-cancer trait pairs were identified (*P* < 5 × 10^−8^) (Additional file 2: Table S3). We failed to identify significant risk loci in the two types of trait pairs “aspirin use-cancer” and “hypnotics use-cancer”. Several shared genomic loci were identified across multiple trait pairs—e.g., rs71658797 was shared between AlcUD-LC and AlcUD-LUSC as well as rs780092, which was shared between AlcUD-BRCA ER^−^ and AlcUD-OCAC (Additional file 2: Table S3). Colocalization analysis using single causal variant assumption (coloc analysis) [87] identified 22 of the 97 (22.68%) potential pleiotropic loci with posterior probability of the shared causal variant hypothesis H4 (PP.H4) values above 0.7, indicating colocalization (Additional file 2: Table S3).

It is noteworthy that some pleiotropic chromosomal regions were found to be shared between different trait pairs. For example, chromosomal regions 1p31.1, 2p23.3, and 15q15.3 were identified as shared genomic regions associated with multiple trait pairs. Specifically, 1p31.1 was linked to both AlcUD-LUSC and AlcUD-LC; 15q15.3 was shared by AlcUD-LUSC and AlcUD-LC; and 2p23.3 was associated with AlcUD-BRCA ER − and AlcUD-OCAC, indicating these regions play a crucial role in the genetic correlations between various cancer types and alcohol use traits (Additional file 2: Table S4).

To detect shared mechanisms of the identified loci, we mapped nearby genes according to the lead SNP in each locus based on its most significant trait association, and under the assumption of its linkage disequilibrium with other SNPs within the loci. Additionally, we performed a multi-marker effect analysis on the GWAS data using MAGMA [67] to explore shared pleiotropic genes across trait pairs. Summarizing the shared loci and genes, circular networks were generated to visualize the pleiotropic architecture of trait pairs (Fig. 3B and Additional file 1: Fig. S1). As exemplified for AlcUD, pairing with BRCA ER −, OCAC, LC, and LUSC was associated each with ≥ 3 loci and ≥ 17 genes (Fig. 3B). Enrichment results from MAGMA indicated that the pleiotropic loci are involved in nicotinic acetylcholine receptor, generation and development of neurons, cell cycle, and transcription regulation (Additional file 1: Fig. S2). Notably, the generation and development of neurons was strongly associated with all trait pairs and cell cycle as well as transcription regulation were detected in most of the trait pairs (Additional file 2: Table S5).

### Tissue and cell-type specificity

**Fig. 4:**
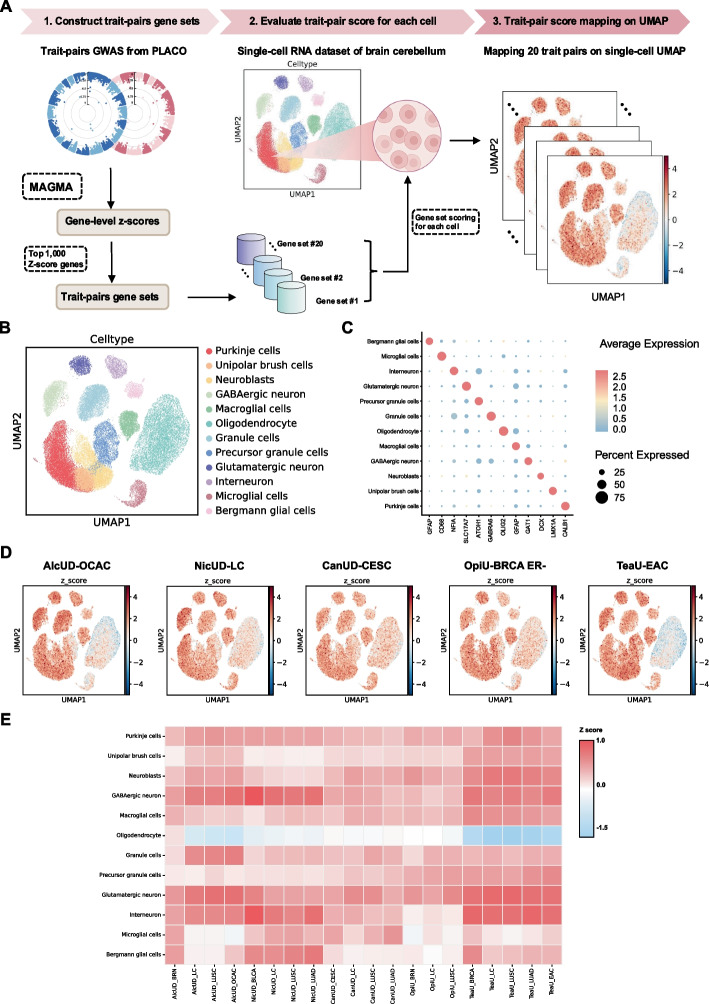
Single-cell specificity inferred from the shared signals between psychoactive substance use and pan-cancer. **A** Schematic representation of the study workflow. **B** UMAP of the cell annotation of scRNAseq dataset of brain cerebellum showing 12 cell types. **C** Dotplot showing specific markers of 12 brain cerebellum cell types. **D** UMAP representation of single-cell disease relevance *z*-scores (scDRS) of the indicated trait pairs. The color represents the disease score, where a darker color indicates a higher score, signifying greater disease enrichment for the specific cell cluster. **E** Heatmap representation of scDRS for 20 indicated trait pairs

Tissue-specific expression analysis using the GTEx database revealed that risk loci were enriched in brain tissue. We further explored 13 different brain region tissues and demonstrated that all trait pairs were significantly associated with the cerebellum (Additional file 2: Table S6). To detect the specificity of different trait pairs at the single-cell level, scDRS analysis was employed (Fig. 4A). A scRNAseq dataset of human brain cerebellum was collected and Purkinje cells, unipolar brush cells, neuroblasts, GABAergic neurons, macroglia, oligodendrocytes, granule cells, precursor granule cells, glutamatergic neurons, interneurons, microglial cells, and Bergmann glial cells were identified based on cell markers (Fig. 4B–C). The results revealed that scDRS of trait pairs were most strongly enriched in GABAergic neurons, granule cells, glutamatergic neurons, and interneurons, and were depleted in oligodendrocytes. These enriched cell subtypes constitute major excitatory and inhibitory circuits of the cerebellum and have been implicated in addictive behaviors, indicating that PSU and cancer trait pairs may share genetic risk through cerebellar neuronal pathways involved in synaptic transmission and behavioral regulation (Fig. 4D–E and Additional file 1: Fig. S3).

### Identification of actionable tissue-specific pleiotropic genes

**Fig. 5:**
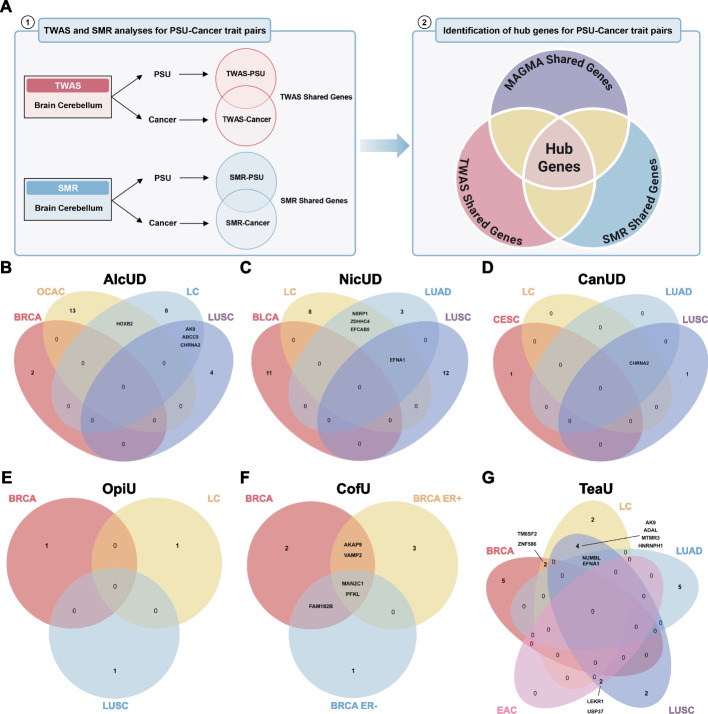
Shared gene signals between different PSU and cancer trait pairs. (**A**) Study design for identifying hub genes shared between different trait pairs. Transcriptome-wide association study (TWAS) and summary-based Mendelian randomization (SMR) analyses were first conducted separately for different PSU and cancer trait pairs using brain cerebellum tissue of GTEx database. The overlap between PSU- and cancer-associated genes in TWAS identified TWAS shared genes, while the overlap in SMR highlighted SMR shared genes with potential causal effects on both traits. Subsequently, the TWAS shared genes, SMR shared genes, and MAGMA shared genes (derived from previous MAGMA analyses of PSU and cancer trait pairs) were integrated to identify hub genes. These hub genes may represent key genetic components underlying the shared biological mechanisms between PSU and cancer. **B**–**G** Venn diagram representation of shared gene signals for alcohol use dependence (AlcUD; **B**), nicotine use dependence (NicUD; **C**), cannabis use disorder (CanUD; **D**), opioid use (OpiU; **E**), coffee use (CofU; **F**), and tea use (TeaU; **G**)

TWAS and SMR analyses were conducted to interrogate gene-tissue effects for each PSU cancer trait pair. TWAS detects the association between genetically predicted mRNA expression and the GWAS trait, whereas SMR evaluates putative causal effects of gene expression on the trait using cis-eQTLs as instrumental variables under the MR method (Fig. 5A). A total of 109 genes identified by MAGMA were subsequently intersected with candidate genes from TWAS and SMR to screen hub genes between different PSU and cancer trait pairs. (Additional file 2: Table S7). We identified 21 pleiotropic genes across multiple trait pairs, with EFNA1 identified as a pleiotropic gene for six trait pairs and CHRNA2 in five trait pairs (Fig. 5B–G; Additional file 2: Table S8).

### The causal association between PSU and cancer

**Fig. 6:**
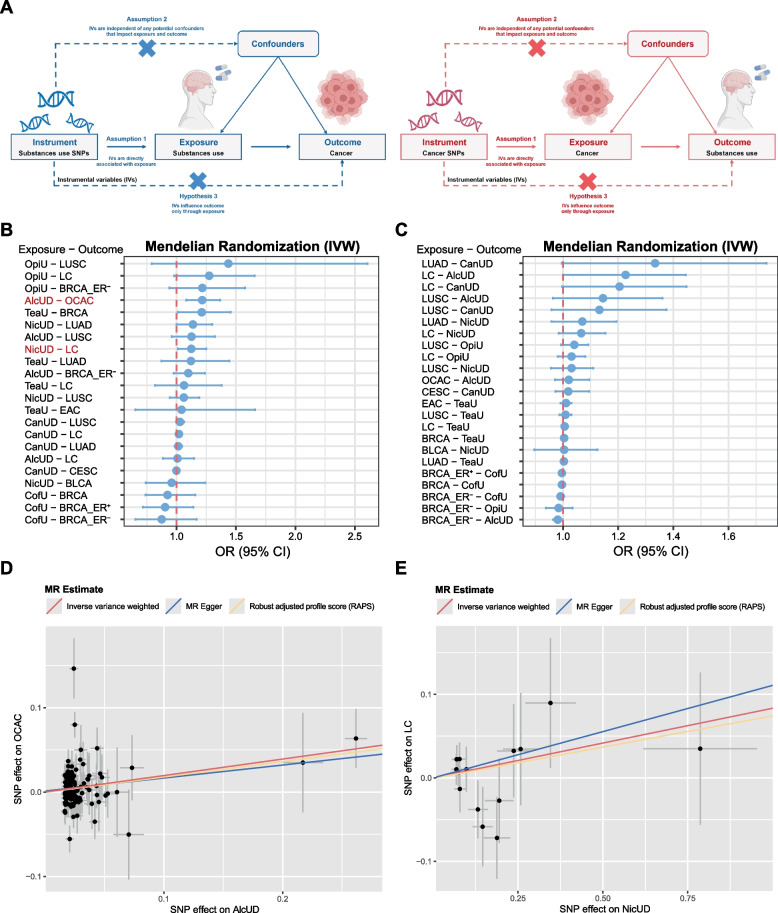
Causal association analysis. **A** Study design of Mendelian randomization (MR) analysis. **B**–**C** The forest plot shows causal associations between PSU and cancer by using MR analysis. **D** Scatter plot shows a significant causal relationship between AlcUD and OCAC risk. **E** Scatter plot shows a significant causal relationship between NicUD and LC risk

MR analysis was applied to assess whether a causal relationship exists between PSU and cancer (Fig. 6A). The IVW-based MR analyses indicated a causal effect of PSU (AlcUD and NicUD) on cancer (OCAC and LC) risk (Fig. 6B–C; Additional file 2: Table S9). The risk of OCAC was observed to increase with higher genetic liability to AlcUD, with the causal effect assessed by the IVW method (OR = 1.216, 95% CI = 1.082–1.366, P = 0.001). Additionally, MR revealed a remarkable causal effect of NicUD on LC risk (IVW, OR = 1.124, 95% CI = 1.010–1.251, *P* = 0.032). Moreover, the scatter plot indicated a favorable concordance among different MR methods (IVW, MR Egger, RAPS, and CAUSE), suggesting a stable and reliable causal inference (Fig. 6D–E, Additional file 2: Table S10). To further explore whether the identified hub genes influence cancer risk through PSU, we employed mediation analysis based on the Two-step MR method. Based on the MR results, AlcUD-OCAC and NicUD-LC were selected for mediation analyses. According to the identified hub genes, HOXB2 was tested for the AlcUD-OCAC pair, while NSRP1, ZDHHC4, and EFCAB5 were tested for the NicUD-LC pair. It was observed that no significant indirect effects were detected across all tested genes (Additional file 2: Table S11), indicating no evidence supporting PSU as a mediator in the causal pathways from hub genes to OCAC or LC.

### Drug target analysis

**Fig. 7:**
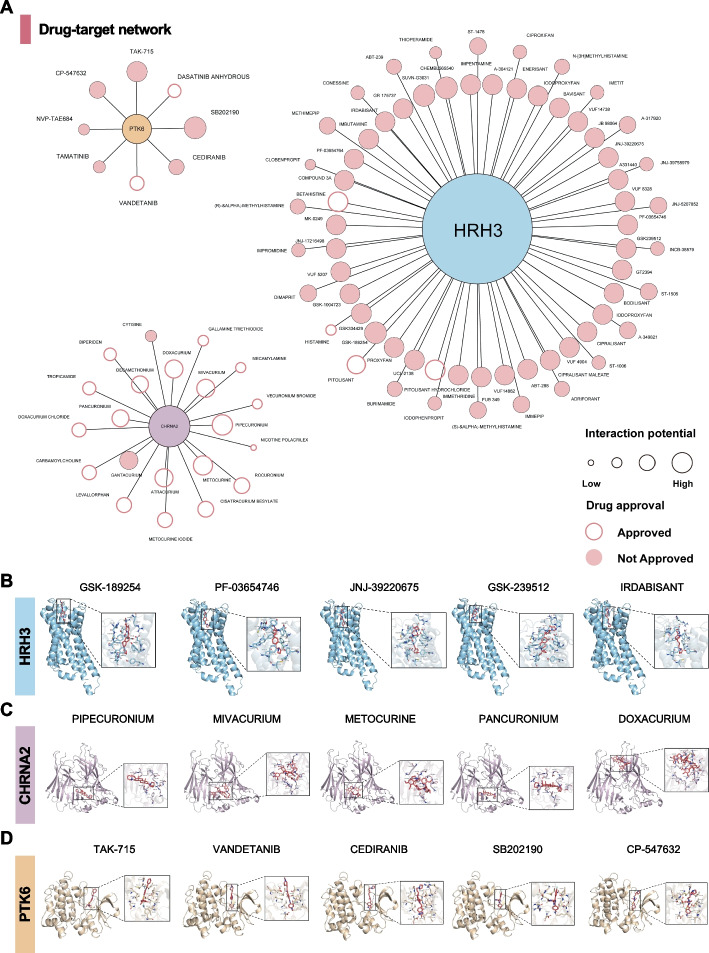
Drug target analysis. **A** Shared genes HRH3, PTK6, and CHRNA2 and their interaction potential with the indicated approved and non-approved drugs (open/filed circles) are depicted. Circle size indicates the interaction potential from low to high.(**B**–**D** Molecular docking analysis of HRH3, CHRNA2, and PTK6 with their potential interacting compounds

To screen potential drugs targeting 21 identified hub genes, we performed a PheWAS using ExPheWas at the gene level to assess whether hub genes have beneficial or deleterious effects on other traits. None of the three genes CHRNA2, HRH3, and PTK6 were significantly associated with other traits (*P* < 5E^−8^ for genomic association; Additional file 2: Table S12). This finding suggested that potential side effects of drugs acting on CHRNA2, HRH3, and PTK6 and the presence of horizontal pleiotropy in these genes are likely to be small. The DGIdb database was used to make predictions of potentially effective interventional drugs on CHRNA2, HRH3, and PTK6 (Fig. 7A and Additional file 2: Table S13).

To verify the prediction results from DGIdb, molecular docking analyses were performed for CHRNA2, HRH3, and PTK6. Docking simulations confirmed stable binding affinities between the predicted compounds and their respective proteins (Additional file 2: Table S14). The top five docking complexes for each of the hub proteins are shown in Fig. 7B–D. The largest number of potential drugs was identified for HRH3, among which GSK-189254 and PF-03654746 exhibited the strongest interactions, as supported by both DGIdb prediction and molecular docking analyses. In addition, PIPECURONIUM was identified as the most promising drug targeting CHRNA2 (Additional file 2: Table S14). Hence, genes identified as central hubs in the shared genetic architecture of PSU and cancers show traits of druggability that may be used in future pre-clinical targeting strategies.

## Discussion

Alcohol and nicotine are estimated to affect up to 30% of individuals worldwide and to annually account for three and seven million deaths, respectively [88]. A substantial proportion of these deaths are related to the induction of malignancies such as lung cancer and HNSCC through carcinogenic compounds present in tobacco products and pro-inflammatory effects of alcohol [15, 16, 89]. However, far less is known about an innate, shared genetic architecture that may coordinately impact on PSU and cancer as (multi-loci) SNPs with mutual interactions and effect sizes [90–92]. Unlike carcinogen-induced mutations, an inherited genetic architecture enhances the likelihood of a trait through coding and non-coding SNP variants that affect gene expression [92, 93]. Underlying genetic architectures can promote comorbidities through pleiotropic genes that impact two or more phenotypes, epistasis and modifier genes influencing disease outcome depending on the genetic background, overlapping pathways, and genetic predispositions that affect individual behavior and responses to environmental cues.

Deeper understanding of genetic architectures provides relevant molecular insight into comorbidities, their interrelations, and may help uncover novel pharmaceutical targets and predictive markers [90, 92, 94, 95]. For example, the PANcreatic Disease ReseArch (PANDoRA) consortium identified 25 susceptibility loci for the occurrence of pancreatic cancer [93], which can be leveraged to understand their contribution to this devastating malignancy. Genetic susceptibility loci identified by GWAS were reported for radiation-induced oral mucositis [96], Barrett´s esophagus and esophageal adenocarcinoma [97], clear cell renal cell carcinoma [98], and ovarian cancer [99], among others. A genetic architecture of liver cirrhosis determined risk-associated loci that were implemented in a polygenic risk score to predict a transition from liver cirrhosis to hepatocellular carcinoma [100]. Potential therapeutic targets to treat alcohol-UD were identified through the integration of proteomic, transcriptomic, and GWAS data [94, 101]. Risk genes related to tobacco use and their association with medical outcomes as diverse as heart disease, pain, and viral infection (i.e., HIV) were described [102, 103]. Most of these findings are very recent and large-scale analysis of PSU and cancer linkage at the level of genetic architecture is yet unavailable to our knowledge.

In our genome-wide case–control meta-analysis, we describe for the first time a pleiotropy underlying the genetic architecture of PSU and cancer linkage. We report a total of 34 shared trait pairs genetically linking lung adeno- and squamous carcinoma, breast carcinoma (ER − and ER +), ovarian, cervical, and esophageal adenocarcinoma with the use of alcohol, nicotine, cannabis, opioids, aspirin, hypnotics, coffee, and tea. Cross-trait pleiotropy analysis using PLACO identified 13 significant risk loci shared across multiple PSU and cancer trait pairs. MAGMA analysis revealed shared biological mechanisms underlying PSU and cancer, including neuronal generation and development, cell cycle regulation, and transcriptional control. Further TWAS and SMR analyses identified 21 shared hub genes, which were subsequently incorporated into drug-target prediction, resulting in a regulatory network comprising three key druggable genes (HRH3, CHRAN2, and PTK6). Finally, MR analysis provided evidence for causal associations between AlcUD and OCAC, as well as between NicUD and LC.

Our analyses revealed remarkable positive global genetic correlations between alcohol dependence, nicotine dependence, and cannabis use disorder and lung cancer, consistent with findings from previous observational studies [104–107]. Further subtype analyses identified differences in genetic correlations across lung cancer subtypes (LUAD, LUSC, and SCLC), thereby extending prior research. Moreover, we observed a positive genetic correlation between cannabis use disorder and cervical cancer. As no observational studies to date have demonstrated a promoting effect of cannabis use disorder on cervical cancer, this novel finding warrants further investigation into the underlying biological mechanisms.

Based on PLACO pleiotropy analysis, we identified a series of genetic risk loci associated with both PSU and cancer. Among these, the 19q13.2 region was of particular interest, as previous studies have demonstrated that this locus may play a crucial role in both PSU and various cancers. For example, the 19q13.2 locus has been reported to be associated with nicotine dependence [108, 109] and has also been linked to increased risks of several cancers, including lung cancer [110–114].

Notably, among all PSU and cancer trait pairs, coffee consumption showed a remarkable negative genetic correlation with breast cancer, in line with previous reports suggesting that coffee intake may reduce the risk of breast cancer [36, 37]. Our finding determined 2p25.3, 15q26.1, and 16q12.2 as shared pleiotropic loci between coffee consumption and breast cancer. These loci have not been reported in previous studies and represent novel findings of the present research. Based on MAGMA analysis, we observed several shared functional pathways between coffee use and breast cancer. Coffee and its bioactive components, such as caffeine, have been shown to regulate multiple nuclear receptors, including the aryl hydrocarbon receptor (AHR) [115, 116], peroxisome proliferator-activated receptors (PPARs) [117], and estrogen receptor (ER) signaling [118]. These receptors are involved in caffeine metabolism and are also closely associated with the development and progression of breast cancer [119–122]. Therefore, genetic variations that affect coffee metabolism or response may influence breast cancer susceptibility by modulating nuclear receptor-mediated transcriptional networks.

Our MR analysis further supports a causal relationship between PSU and cancer risk. Specifically, results based on the IVW method showed that increased genetic liability to AlcUD was significantly associated with a higher risk of OCAC, while NicUD exhibited a significant causal effect on LC, which is consistent with previous epidemiological studies [105, 123]. The concordant results across multiple MR methods (IVW, MR-Egger, RAPS, and CAUSE) further strengthen the robustness of our causal inference. The causal links identified in this study highlight the importance of PSU as a modifiable behavioral risk factor in cancer prevention.

Pleiotropy is a central aspect of comorbidities, which we mapped to loci and SNPs acting on hub genes in GABAergic neurons, granule cells, glutamatergic neurons, and interneurons of the brain cerebellum. The cerebellum emerged as an important factor in cancers through cognitive, emotional, and systemic regulation of the immune system, response to (chronic) stress, inflammation, angiogenesis, shaping of the tumor microenvironment, and through reward mechanisms and addiction [124–132]. Immunosurveillance and inflammation are regulated through cerebellum-derived neural pathways including the hypothalamic–pituitary–adrenal (HPA) pathway. These regulatory mechanisms are usurped by tumors for purposes of immune evasion [125, 133], leading to enhanced cortisol and stress hormones that have immunosuppressive functions supporting tumor outgrowth [134, 135]. The cerebellum further regulates a higher-order cognitive and emotional circuitry that promotes depression and anxiety when dysfunctional. Thereby, it negatively impacts on the patients’ outcome, compliance to treatment, and predisposition to addiction [136–138]. Based on all these implications in cancer outcome, approaches to re-install functional cerebellar activities are under development, including transcranial magnetic stimulation (TMS), cerebellar neurofeedback, and cognitive rehabilitation to revert dysfunctions [127].

In the present study, numerous approved and non-approved drugs were identified by using the GDIdb database for hub genes *CHRNA2*, *HRH3*, and *PTK6* that are pleiotropic genes affecting several PSU-cancer trait pairs. *CHRNA2* encodes the α2 subunit of neuronal nicotinic acetylcholine receptors (nAChR), which are ion channels activated upon binding of acetylcholine or nicotine. Knockout of *CHRNA2* in female mice resulted in an increased cued fear conditioning following acute nicotine administration [139], whereas hypersensitive CHRNA2 hampered contextual fear conditioning [140]. *CHRNA2* is affected by a lead SNP determined in a large-scale GWAS of cannabis UD and a missense mutation from Threonine 22 to Isoleucine is associated with addiction to nicotine [48, 141, 142]. Enhanced expression of *CHRNA2* in prostate cancer correlated with poor response to immune checkpoint inhibition [143], and SNPs in the *CHRNA2* locus predict susceptibility to lung cancer [50]. HRH3 is up-regulated in the pre-frontal cortex during social isolation-induced chronic stress [144], in lung cancer stem cells [145], and can be targeted with numerous drugs, according to the GDIdb and to reports of its inhibition in conjunction with alcohol UD [146]. In line with the effects of CHRNA2, HRH3 was shown to exert a negative regulation on peripheral immune cells following inflammatory signals [147]. Enhanced PTK6 expression correlates with poor outcomes and/or drives oncogenic progression of renal cell, hepatocellular, breast, ovarian, and prostate carcinoma [148–153]. Accordingly, inhibitory targeting of PTK6 has been interrogated in multiple cancers [154]. Furthermore, PTK6 has been recently associated with pathological drinking behavior and schizophrenia, major depression, and bipolar disorder [155].

Thus, our findings suggest an implication of the PSU-cancer genetic architecture in influencing behavioral and non-behavioral aspects with repercussions on substance use and cancer [156–158]. Inhibition of hub genes with pleiotropic effects in PSU-cancer trait pairs represents a potential avenue for future research into preventing comorbidities entailed by trait linkage. This is particularly of interest with respect to cannabinoids and opioids, as they are in use for the treatment of cancer patients to enhance appetite and reduce disease-entailed pain. Furthermore, defined SNPs and hub gene expressions may be investigated as potential markers to identify patients suffering from psychoactive drug abuse or from cancer who are at increased risk to develop respectively linked illnesses. Such stratification could be explored as a framework for improved personalized medicine and early detection to improve the prognosis of patients.

### Limitations

The genetic architecture of illnesses and their linkage depend on genetic ancestry, which is not covered in an unbiased manner in current GWAS, as studies include mostly individuals of European ancestry [92, 159, 160]. Hence, future studies are needed in non-European populations. Further, a medical benefit of targeting hub genes associated with PSU-cancer trait pairs remains to be validated in experimental and clinical settings, which represents a challenging task.

## Conclusions

This large post-GWAS study identifies a shared genetic architecture between psychoactive substance use (PSU) and cancer risk. The findings highlight genes linked to the brain’s reward system and social behavior, primarily expressed in the cerebellum, including three druggable targets. These results offer new insights into an underexplored genetic basis that predisposes individuals to both PSU and cancer, potentially paving the way for combinatorial treatment approaches for these common comorbidities.

## Supplementary Information

Additional file 1. Figures S1–S3.

Additional file 2. Tables S1–S14. Table S1. Data sources. Table S2. Genetic correlation analysis conducted by LDSC and HDL. Table S3. Shared pleiotropic loci identified by PLACO. Table S4. Shared pleiotropic loci among different trait-pairs. Table S5. MAGMA Gene-set analysis. Table S6. MAGMA tissue-specific analysis. Table S7. Tissue-specific pleiotropic genes. Table S8. Hub pleiotropic genes across multiple trait-pairs. Table S9. Causal association analysis by MR method. Table S10. Summary of CAUSE results. Table S11. Two-step Mendelian randomization mediation analysis. Table S12. Phenome-Wide Association Study. Table S13. Drug target analysis by DGIdb database. Table S14. Molecular docking analysis.

Additional file 3. STROBE-MR-checklist.

## Data Availability

Data are available in public, open access repositories corresponding to the original studies (e.g., GWAS catalog). All codes and R-packages used in the study are publicly available and have been disclosed in Methods or are available from the corresponding authors on reasonable request.

## Abbreviations

AlcUD: Alcohol use dependence
BCAC: Breast Cancer Association Consortium
BLCA: Bladder cancer
BRCA: Breast cancer
CanUD: Cannabis use disorder
CHRNA2: Cholinergic receptor nicotinic alpha 2
CoFU: Coffee intake
DGIdb: Drug-gene interactions database
ER: Estrogen receptor
FUMA: Functional mapping and annotation
GSCAN: GWAS and Sequencing Consortium of Alcohol and Nicotine Use
GTEX: Genotype-tissue expression
GWAS: Genome-wide association study
GWTSEA: Genome-wide tissue-specific enrichment analysis
HDL: High-definition likelihood
HRH3: Histamine receptor H3
HPA: Hypothalamic-pituitary-adrenal
HypU: Hypnotics use
ILCCO: International Lung Cancer Consortium
LC: Lung cancer
LDSC: Linkage disequilibrium score
LGG: Low-grade glioma
LUAD: Lung adenocarcinoma
LUSC: Lung squamous cell carcinoma
MAGMA: Multi-marker analysis of genomic annotation
MC: Monte Carlo
MELA: Melanoma
MsigDB: Molecular signatures database
NicUD: Nicotine use dependence
OCAC: Ovarian Cancer Association Consortium
OpiU: Opiod use
PANDoRA: PANcreatic Disease ReseArch consortium
PCG: Psychiatric genomics consortium
PLACO: Pleiotropic analysis under composite null
PP: Posterior probability
PRACTICAL: Prostate Cancer Association Group to Investigate Cancer Associated Alterations in the Genome
PRAD: Prostate cancer
PSU: Psychoactive substance use
PTK6: Protein tyrosine kinase 6
RCC: Renal cell carcinoma
scDRS: Single-cell disease relevance score
SCLC: Small cell lung cancer
scRNAseq: Single-cell RNA sequencing
SMR: Summary data-based Mendelian randomization
SNP: Single nucleotide polymorphism
SUD: Substance use disorders
TeaU: Tea intake
TMS: Transcranial magnetic stimulation
TWAS: Transcriptome-wide association study
UCEC: Uterine corpus endometrial cancer

## References

[1] Deak JD, Johnson EC. Genetics of substance use disorders: a review. Psychol Med. 2021;51(13):2189–200. 33879270 10.1017/S0033291721000969 PMC8477224

[2] Moussas GI, Papadopoulou AG. Substance abuse and cancer. Psychiatriki. 2017;28(3):234–41. 29072187 10.22365/jpsych.2017.283.234

[3] Sanchez-Roige S, Kember RL, Agrawal A. Substance use and common contributors to morbidity: a genetics perspective. EBioMedicine. 2022;83:104212. 35970022 10.1016/j.ebiom.2022.104212 PMC9399262

[4] Gerring ZF, Thorp JG, Treur JL, Verweij KJH, Derks EM. The genetic landscape of substance use disorders. Mol Psychiatry. 2024. 10.1038/s41380-024-02547-z. 38811691 10.1038/s41380-024-02547-z PMC11541208

[5] Wise RA, Jordan CJ. Dopamine, behavior, and addiction. J Biomed Sci. 2021;28(1):83. 34852810 10.1186/s12929-021-00779-7 PMC8638539

[6] Auger N, Paradis G, Low N, Ayoub A, He S, Potter BJ. Cannabis use disorder and the future risk of cardiovascular disease in parous women: a longitudinal cohort study. BMC Med. 2020;18(1):328. 33208143 10.1186/s12916-020-01804-6 PMC7677785

[7] Nierengarten MB. Cancer Statistics 2024: deaths drop, incidences increase, prevention needed. Cancer. 2024;130(11):1904. 38757629 10.1002/cncr.35347

[8] Siegel RL, Giaquinto AN, Jemal A. Cancer statistics, 2024. CA Cancer J Clin. 2024;74(1):12–49. 38230766 10.3322/caac.21820

[9] Fisher ML, Pauly JR, Froeliger B, Turner JR. Translational research in nicotine addiction. Cold Spring Harb Perspect Med. 2021. 10.1101/cshperspect.a039776. 32513669 10.1101/cshperspect.a039776 PMC8168530

[10] Rehm J, Assanangkornchai S, Hendershot CS, Franklin A, Neufeld M, Hassan AS, et al. Alcohol use disorders. Lancet. 2025;406(10516):2269–81. 41077052 10.1016/S0140-6736(25)01496-5

[11] Collaborators GBDA. Alcohol use and burden for 195 countries and territories, 1990-2016: a systematic analysis for the Global Burden of Disease Study 2016. Lancet. 2018;392(10152):1015–35. 30146330 10.1016/S0140-6736(18)31310-2 PMC6148333

[12] Anderson BO, Berdzuli N, Ilbawi A, Kestel D, Kluge HP, Krech R, et al. Health and cancer risks associated with low levels of alcohol consumption. Lancet Public Health. 2023;8(1):e6–7. 36603913 10.1016/S2468-2667(22)00317-6 PMC9831798

[13] Brennan P, Hainaut P, Boffetta P. Genetics of lung-cancer susceptibility. Lancet Oncol. 2011;12(4):399–408. 20951091 10.1016/S1470-2045(10)70126-1

[14] Mody MD, Rocco JW, Yom SS, Haddad RI, Saba NF. Head and neck cancer. Lancet. 2021;398(10318):2289–99. 34562395 10.1016/S0140-6736(21)01550-6

[15] Leemans CR, Snijders PJF, Brakenhoff RH. The molecular landscape of head and neck cancer. Nat Rev Cancer. 2018;18(5):269–82. 29497144 10.1038/nrc.2018.11

[16] Johnson DE, Burtness B, Leemans CR, Lui VWY, Bauman JE, Grandis JR. Head and neck squamous cell carcinoma. Nat Rev Dis Primers. 2020;6(1):92. 33243986 10.1038/s41572-020-00224-3 PMC7944998

[17] Hartz SM, Bierut LJ. Genetics of addictions. Psychiatr Clin North Am. 2010;33(1):107–24. 20159342 10.1016/j.psc.2009.10.003 PMC3047504

[18] Hall W, Lynskey M. Assessing the public health impacts of legalizing recreational cannabis use: the US experience. World Psychiatry. 2020;19(2):179–86. 32394566 10.1002/wps.20735 PMC7215066

[19] Johnson JR, Burnell-Nugent M, Lossignol D, Ganae-Motan ED, Potts R, Fallon MT. Multicenter, double-blind, randomized, placebo-controlled, parallel-group study of the efficacy, safety, and tolerability of THC:CBD extract and THC extract in patients with intractable cancer-related pain. J Pain Symptom Manage. 2010;39(2):167–79. 19896326 10.1016/j.jpainsymman.2009.06.008

[20] Good P, Haywood A, Gogna G, Martin J, Yates P, Greer R, et al. Oral medicinal cannabinoids to relieve symptom burden in the palliative care of patients with advanced cancer: a double-blind, placebo controlled, randomised clinical trial of efficacy and safety of cannabidiol (CBD). BMC Palliat Care. 2019;18(1):110. 31810437 10.1186/s12904-019-0494-6 PMC6898965

[21] Cuomo RE. Cannabis use disorder and five-year risk of oral cancer in a multicenter clinical cohort. Prev Med Rep. 2025;57:103185. 40746396 10.1016/j.pmedr.2025.103185 PMC12311940

[22] Mohammed T, Yu J, Qiao Y, Kim Y, Mortensen E, Swede H, et al. Marijuana use may be associated with reduced prevalence of prostate cancer: a national survey on drug use and health study from United States of America. Biomedicines. 2024. 10.3390/biomedicines12051008. 38790970 10.3390/biomedicines12051008 PMC11118915

[23] Paice JA, Bohlke K, Barton D, Craig DS, El-Jawahri A, Hershman DL, et al. Use of Opioids for Adults With Pain From Cancer or Cancer Treatment: ASCO Guideline. J Clin Oncol. 2023;41(4):914–30. 36469839 10.1200/JCO.22.02198

[24] Tripolt S, Neubauer HA, Knab VM, Elmer DP, Aberger F, Moriggl R, et al. Opioids drive breast cancer metastasis through the delta-opioid receptor and oncogenic STAT3. Neoplasia. 2021;23(2):270–9. 33465556 10.1016/j.neo.2020.12.011 PMC7815495

[25] Sun M, Lin JA, Chang CL, Wu SY, Zhang J. Association between long-term opioid use and cancer risk in patients with chronic pain: a propensity score-matched cohort study. Br J Anaesth. 2022;129(1):84–91. 35597621 10.1016/j.bja.2022.04.014

[26] Dai S, Zhang X, Zhang P, Zheng X, Pang Q. Fentanyl inhibits acute myeloid leukemia differentiated cells and committed progenitors via opioid receptor-independent suppression of Ras and STAT5 pathways. Fundam Clin Pharmacol. 2021;35(1):174–83. 32564393 10.1111/fcp.12581

[27] Eisenstein M. Tea’s value as a cancer therapy is steeped in uncertainty. Nature. 2019;566(7742):S6–7. 30728516 10.1038/d41586-019-00397-2

[28] Butt MS, Sultan MT. Coffee and its consumption: benefits and risks. Crit Rev Food Sci Nutr. 2011;51(4):363–73. 21432699 10.1080/10408390903586412

[29] Poole R, Kennedy OJ, Roderick P, Fallowfield JA, Hayes PC, Parkes J. Coffee consumption and health: umbrella review of meta-analyses of multiple health outcomes. BMJ. 2017;359:j5024. 29167102 10.1136/bmj.j5024 PMC5696634

[30] Ludwig IA, Clifford MN, Lean ME, Ashihara H, Crozier A. Coffee: biochemistry and potential impact on health. Food Funct. 2014;5(8):1695–717. 24671262 10.1039/c4fo00042k

[31] Iwasaki M, Mizusawa J, Kasuga Y, Yokoyama S, Onuma H, Nishimura H, et al. Green tea consumption and breast cancer risk in Japanese women: a case-control study. Nutr Cancer. 2014;66(1):57–67. 24274352 10.1080/01635581.2014.847963

[32] Sheerah H, Keyang L, Eshak ES, Cui R, Shirai K, Muraki I, et al. Association of tea consumption and the risk of gastric cancer in Japanese adults: the Japan Collaborative Cohort Study. BMJ Open. 2020;10(10):e038243. 33028558 10.1136/bmjopen-2020-038243 PMC7539605

[33] Kim TL, Jeong GH, Yang JW, Lee KH, Kronbichler A, van der Vliet HJ, et al. Tea consumption and risk of cancer: an umbrella review and meta-analysis of observational studies. Adv Nutr. 2020;11(6):1437–52. 32667980 10.1093/advances/nmaa077 PMC7666907

[34] Pounis G, Tabolacci C, Costanzo S, Cordella M, Bonaccio M, Rago L, et al. Reduction by coffee consumption of prostate cancer risk: evidence from the Moli-sani cohort and cellular models. Int J Cancer. 2017;141(1):72–82. 28436066 10.1002/ijc.30720

[35] Rhee J, Loftfield E, Freedman ND, Liao LM, Sinha R, Purdue MP. Coffee consumption and risk of renal cell carcinoma in the NIH-AARP diet and health study. Int J Epidemiol. 2021;50(5):1473–81. 33624757 10.1093/ije/dyab011 PMC8783596

[36] Bhoo-Pathy N, Peeters PH, Uiterwaal CS, Bueno-de-Mesquita HB, Bulgiba AM, Bech BH, et al. Coffee and tea consumption and risk of pre- and postmenopausal breast cancer in the European Prospective Investigation into Cancer and Nutrition (EPIC) cohort study. Breast Cancer Res. 2015;17(1):15. 25637171 10.1186/s13058-015-0521-3 PMC4349221

[37] Lafranconi A, Micek A, De Paoli P, Bimonte S, Rossi P, Quagliariello V, et al. Coffee intake decreases risk of postmenopausal breast cancer: a dose-response meta-analysis on prospective cohort studies. Nutrients. 2018. 10.3390/nu10020112. 29360766 10.3390/nu10020112 PMC5852688

[38] Wang A, Wang S, Zhu C, Huang H, Wu L, Wan X, et al. Coffee and cancer risk: A meta-analysis of prospective observational studies. Sci Rep. 2016;6:33711. 27665923 10.1038/srep33711 PMC5036059

[39] Zhang Y, Ma C, Zhao L, Mucci LA, Giovannucci EL. Decaffeinated coffee consumption and risk of total and site-specific cancer. Ann Oncol. 2025;36(7):819–31. 40180122 10.1016/j.annonc.2025.03.018 PMC12167682

[40] Yu X, Chen Y, Chen J, Fan Y, Lu H, Wu D, et al. Shared genetic architecture between autoimmune disorders and B-cell acute lymphoblastic leukemia: insights from large-scale genome-wide cross-trait analysis. BMC Med. 2024;22(1):161. 38616254 10.1186/s12916-024-03385-0 PMC11017616

[41] You D, Wu Y, Lu M, Shao F, Tang Y, Liu S, et al. A genome-wide cross-trait analysis characterizes the shared genetic architecture between lung and gastrointestinal diseases. Nat Commun. 2025;16(1):3032. 40155373 10.1038/s41467-025-58248-w PMC11953465

[42] Wang S, Liu H, Yang Y, Wang Q, Zhang C, Zhang S, et al. Investigating the shared genetic architecture between adiposity measures and obesity-related cancers. Brief Bioinform. 2025. 10.1093/bib/bbaf439. 40874817 10.1093/bib/bbaf439 PMC12392268

[43] Koller D, Friligkou E, Stiltner B, Pathak GA, Lokhammer S, Levey DF, et al. Pleiotropy and genetically inferred causality linking multisite chronic pain to substance use disorders. Mol Psychiatry. 2024;29(7):2021–30. 38355787 10.1038/s41380-024-02446-3 PMC11324857

[44] Malone SG, Davis CN, Piserchia Z, Setzer MR, Toikumo S, Zhou H, et al. Alcohol use disorder and body mass index show genetic pleiotropy and shared neural associations. Nat Hum Behav. 2025;9(5):1056–66. 40164914 10.1038/s41562-025-02148-y PMC13007225

[45] Lai RY, Su MH, Lin YF, Chen CY, Pan YJ, Hsiao PC, et al. Relationship between mood disorders and substance involvement and the shared genetic liabilities: a population-based study in Taiwan. J Affect Disord. 2024;345:168–76. 37879417 10.1016/j.jad.2023.10.141

[46] Walters RK, Polimanti R, Johnson EC, McClintick JN, Adams MJ, Adkins AE, et al. Transancestral GWAS of alcohol dependence reveals common genetic underpinnings with psychiatric disorders. Nat Neurosci. 2018;21(12):1656–69. 30482948 10.1038/s41593-018-0275-1 PMC6430207

[47] Watanabe K, Stringer S, Frei O, Umicevic Mirkov M, de Leeuw C, Polderman TJC, et al. A global overview of pleiotropy and genetic architecture in complex traits. Nat Genet. 2019;51(9):1339–48. 31427789 10.1038/s41588-019-0481-0

[48] Johnson EC, Demontis D, Thorgeirsson TE, Walters RK, Polimanti R, Hatoum AS, et al. A large-scale genome-wide association study meta-analysis of cannabis use disorder. Lancet Psychiatry. 2020;7(12):1032–45. 33096046 10.1016/S2215-0366(20)30339-4 PMC7674631

[49] Michailidou K, Lindstrom S, Dennis J, Beesley J, Hui S, Kar S, et al. Association analysis identifies 65 new breast cancer risk loci. Nature. 2017;551(7678):92–4. 29059683 10.1038/nature24284 PMC5798588

[50] McKay JD, Hung RJ, Han Y, Zong X, Carreras-Torres R, Christiani DC, et al. Large-scale association analysis identifies new lung cancer susceptibility loci and heterogeneity in genetic susceptibility across histological subtypes. Nat Genet. 2017;49(7):1126–32. 28604730 10.1038/ng.3892 PMC5510465

[51] Rashkin SR, Graff RE, Kachuri L, Thai KK, Alexeeff SE, Blatchins MA, et al. Pan-cancer study detects genetic risk variants and shared genetic basis in two large cohorts. Nat Commun. 2020;11(1):4423. 32887889 10.1038/s41467-020-18246-6 PMC7473862

[52] Koyanagi YN, Nakatochi M, Namba S, Oze I, Charvat H, Narita A, et al. Genetic architecture of alcohol consumption identified by a genotype-stratified GWAS and impact on esophageal cancer risk in Japanese people. Sci Adv. 2024;10(4):eade2780. 38277453 10.1126/sciadv.ade2780 PMC10816704

[53] Quach BC, Bray MJ, Gaddis NC, Liu M, Palviainen T, Minica CC, et al. Expanding the genetic architecture of nicotine dependence and its shared genetics with multiple traits. Nat Commun. 2020;11(1):5562. 33144568 10.1038/s41467-020-19265-z PMC7642344

[54] Wu Y, Byrne EM, Zheng Z, Kemper KE, Yengo L, Mallett AJ, et al. Genome-wide association study of medication-use and associated disease in the UK Biobank. Nat Commun. 2019;10(1):1891. 31015401 10.1038/s41467-019-09572-5 PMC6478889

[55] Sudlow C, Gallacher J, Allen N, Beral V, Burton P, Danesh J, et al. UK biobank: an open access resource for identifying the causes of a wide range of complex diseases of middle and old age. PLoS Med. 2015;12(3):e1001779. 25826379 10.1371/journal.pmed.1001779 PMC4380465

[56] Kurki MI, Karjalainen J, Palta P, Sipila TP, Kristiansson K, Donner KM, et al. FinnGen provides genetic insights from a well-phenotyped isolated population. Nature. 2023;613(7944):508–18. 36653562 10.1038/s41586-022-05473-8 PMC9849126

[57] Melin BS, Barnholtz-Sloan JS, Wrensch MR, Johansen C, Il’yasova D, Kinnersley B, et al. Genome-wide association study of glioma subtypes identifies specific differences in genetic susceptibility to glioblastoma and non-glioblastoma tumors. Nat Genet. 2017;49(5):789–94. 28346443 10.1038/ng.3823 PMC5558246

[58] Gharahkhani P, Fitzgerald RC, Vaughan TL, Palles C, Gockel I, Tomlinson I, et al. Genome-wide association studies in oesophageal adenocarcinoma and Barrett’s oesophagus: a large-scale meta-analysis. Lancet Oncol. 2016;17(10):1363–73. 27527254 10.1016/S1470-2045(16)30240-6 PMC5052458

[59] Gharahkhani P, Fitzgerald RC, Vaughan TL, Palles C, Gockel I, Tomlinson I, et al. Genome-wide association studies in oesophageal adenocarcinoma and Barrett's oesophagus: a large-scale meta-analysis. https://www.ebi.ac.uk/gwas/studies/GCST003739. (2016). 10.1016/S1470-2045(16)30240-6 PMC5052458 27527254

[60] Schumacher FR, Al Olama AA, Berndt SI, Benlloch S, Ahmed M, Saunders EJ, et al. Association analyses of more than 140,000 men identify 63 new prostate cancer susceptibility loci. Nat Genet. 2018;50(7):928–36. 29892016 10.1038/s41588-018-0142-8 PMC6568012

[61] Schumacher FR, Al Olama AA, Berndt SI, Benlloch S, Ahmed M, Saunders EJ, et al. Association analyses of more than 140,000 men identify 63 new prostate cancer susceptibility loci. https://www.ebi.ac.uk/gwas/studies/GCST00608. (2018). 10.1038/s41588-018-0142-8 PMC6568012 29892016

[62] Phelan CM, Kuchenbaecker KB, Tyrer JP, Kar SP, Lawrenson K, Winham SJ, et al. Identification of 12 new susceptibility loci for different histotypes of epithelial ovarian cancer. Nat Genet. 2017;49(5):680–91. 28346442 10.1038/ng.3826 PMC5612337

[63] Bulik-Sullivan B, Finucane HK, Anttila V, Gusev A, Day FR, Loh PR, et al. An atlas of genetic correlations across human diseases and traits. Nat Genet. 2015;47(11):1236–41. 26414676 10.1038/ng.3406 PMC4797329

[64] Ning Z, Pawitan Y, Shen X. High-definition likelihood inference of genetic correlations across human complex traits. Nat Genet. 2020;52(8):859–64. 32601477 10.1038/s41588-020-0653-y

[65] Ray D, Chatterjee N. A powerful method for pleiotropic analysis under composite null hypothesis identifies novel shared loci between type 2 diabetes and prostate cancer. PLoS Genet. 2020;16(12):e1009218. 33290408 10.1371/journal.pgen.1009218 PMC7748289

[66] Watanabe K, Taskesen E, van Bochoven A, Posthuma D. Functional mapping and annotation of genetic associations with FUMA. Nat Commun. 2017;8(1):1826. 29184056 10.1038/s41467-017-01261-5 PMC5705698

[67] de Leeuw CA, Mooij JM, Heskes T, Posthuma D. MAGMA: generalized gene-set analysis of GWAS data. PLoS Comput Biol. 2015;11(4):e1004219. 25885710 10.1371/journal.pcbi.1004219 PMC4401657

[68] Subramanian A, Tamayo P, Mootha VK, Mukherjee S, Ebert BL, Gillette MA, et al. Gene set enrichment analysis: a knowledge-based approach for interpreting genome-wide expression profiles. Proc Natl Acad Sci U S A. 2005;102(43):15545–50. 16199517 10.1073/pnas.0506580102 PMC1239896

[69] Carithers LJ, Ardlie K, Barcus M, Branton PA, Britton A, Buia SA, et al. A novel approach to high-quality postmortem tissue procurement: the GTEx project. Biopreserv Biobank. 2015;13(5):311–9. 26484571 10.1089/bio.2015.0032 PMC4675181

[70] Foley CN, Staley JR, Breen PG, Sun BB, Kirk PDW, Burgess S, et al. A fast and efficient colocalization algorithm for identifying shared genetic risk factors across multiple traits. Nat Commun. 2021;12(1):764. 33536417 10.1038/s41467-020-20885-8 PMC7858636

[71] Zhang MJ, Hou K, Dey KK, Sakaue S, Jagadeesh KA, Weinand K, et al. Polygenic enrichment distinguishes disease associations of individual cells in single-cell RNA-seq data. Nat Genet. 2022;54(10):1572–80. 36050550 10.1038/s41588-022-01167-z PMC9891382

[72] Gusev A, Ko A, Shi H, Bhatia G, Chung W, Penninx BW, et al. Integrative approaches for large-scale transcriptome-wide association studies. Nat Genet. 2016;48(3):245–52. 26854917 10.1038/ng.3506 PMC4767558

[73] Zhu Z, Zhang F, Hu H, Bakshi A, Robinson MR, Powell JE, et al. Integration of summary data from GWAS and eQTL studies predicts complex trait gene targets. Nat Genet. 2016;48(5):481–7. 27019110 10.1038/ng.3538

[74] Genomes Project C, Auton A, Brooks LD, Durbin RM, Garrison EP, Kang HM, et al. A global reference for human genetic variation. Nature. 2015;526(7571):68–74. 26432245 10.1038/nature15393 PMC4750478

[75] Burgess S, Thompson SG, Collaboration CCG. Avoiding bias from weak instruments in Mendelian randomization studies. Int J Epidemiol. 2011;40(3):755–64. 21414999 10.1093/ije/dyr036

[76] Burgess S, Butterworth A, Thompson SG. Mendelian randomization analysis with multiple genetic variants using summarized data. Genet Epidemiol. 2013;37(7):658–65. 24114802 10.1002/gepi.21758 PMC4377079

[77] Burgess S, Thompson SG. Interpreting findings from Mendelian randomization using the MR-Egger method. Eur J Epidemiol. 2017;32(5):377–89. 28527048 10.1007/s10654-017-0255-x PMC5506233

[78] Hemani G, Bowden J, Davey Smith G. Evaluating the potential role of pleiotropy in Mendelian randomization studies. Hum Mol Genet. 2018;27(R2):R195–208. 29771313 10.1093/hmg/ddy163 PMC6061876

[79] Morrison J, Knoblauch N, Marcus JH, Stephens M, He X. Mendelian randomization accounting for correlated and uncorrelated pleiotropic effects using genome-wide summary statistics. Nat Genet. 2020;52(7):740–7. 32451458 10.1038/s41588-020-0631-4 PMC7343608

[80] Bowden J, Del Greco MF MF, Minelli C, Davey Smith G, Sheehan NA, Thompson JR. Assessing the suitability of summary data for two-sample Mendelian randomization analyses using MR-Egger regression: the role of the I2 statistic. Int J Epidemiol. 2016;45(6):1961–74. 27616674 10.1093/ije/dyw220 PMC5446088

[81] Carter AR, Sanderson E, Hammerton G, Richmond RC, Davey Smith G, Heron J, et al. Mendelian randomisation for mediation analysis: current methods and challenges for implementation. Eur J Epidemiol. 2021;36(5):465–78. 33961203 10.1007/s10654-021-00757-1 PMC8159796

[82] Legault MA, Perreault LL, Tardif JC, Dube MP. ExPheWas: a platform for cis-Mendelian randomization and gene-based association scans. Nucleic Acids Res. 2022;50(W1):W305–11. 35474380 10.1093/nar/gkac289 PMC9252780

[83] Cannon M, Stevenson J, Stahl K, Basu R, Coffman A, Kiwala S, et al. DGIdb 5.0: rebuilding the drug-gene interaction database for precision medicine and drug discovery platforms. Nucleic Acids Res. 2024;52(D1):D1227-D35. 10.1093/nar/gkad1040 PMC10767982 37953380

[84] Eberhardt J, Santos-Martins D, Tillack AF, Forli S. AutoDock Vina 1.2.0: new docking methods, expanded force field, and Python bindings. J Chem Inf Model. 2021;61(8):3891–8. 34278794 10.1021/acs.jcim.1c00203 PMC10683950

[85] Giambartolomei C, Vukcevic D, Schadt EE, Franke L, Hingorani AD, Wallace C, et al. Bayesian test for colocalisation between pairs of genetic association studies using summary statistics. PLoS Genet. 2014;10(5):e1004383. 24830394 10.1371/journal.pgen.1004383 PMC4022491

[86] Yavorska OO, Burgess S. MendelianRandomization: an R package for performing Mendelian randomization analyses using summarized data. Int J Epidemiol. 2017;46(6):1734–9. 28398548 10.1093/ije/dyx034 PMC5510723

[87] Wallace C, Cutler AJ, Pontikos N, Pekalski ML, Burren OS, Cooper JD, et al. Dissection of a complex disease susceptibility region using a Bayesian stochastic search approach to fine mapping. PLoS Genet. 2015;11(6):e1005272. 26106896 10.1371/journal.pgen.1005272 PMC4481316

[88] Grant BF, Saha TD, Ruan WJ, Goldstein RB, Chou SP, Jung J, et al. <article-title update="added"> Epidemiology of *DSM-5* Drug Use Disorder : Results From the National Epidemiologic Survey on Alcohol and Related Conditions–III. JAMA Psychiatr. 2016;73(1):39–47. 10.1001/jamapsychiatry.2015.2132 PMC5062605 26580136

[89] Meyer ML, Peters S, Mok TS, Lam S, Yang PC, Aggarwal C, et al. Lung cancer research and treatment: global perspectives and strategic calls to action. Ann Oncol. 2024. 10.1016/j.annonc.2024.10.006. 39413875 10.1016/j.annonc.2024.10.006

[90] Timpson NJ, Greenwood CMT, Soranzo N, Lawson DJ, Richards JB. Genetic architecture: the shape of the genetic contribution to human traits and disease. Nat Rev Genet. 2018;19(2):110–24. 29225335 10.1038/nrg.2017.101

[91] Hormozdiari F, Gazal S, van de Geijn B, Finucane HK, Ju CJ, Loh PR, et al. Leveraging molecular quantitative trait loci to understand the genetic architecture of diseases and complex traits. Nat Genet. 2018;50(7):1041–7. 29942083 10.1038/s41588-018-0148-2 PMC6030458

[92] Lappalainen T, Li YI, Ramachandran S, Gusev A. Genetic and molecular architecture of complex traits. Cell. 2024;187(5):1059–75. 38428388 10.1016/j.cell.2024.01.023 PMC10977002

[93] Campa D, Gentiluomo M, Stein A, Aoki MN, Oliverius M, Vodickova L, et al. The PANcreatic Disease ReseArch (PANDoRA) consortium: Ten years’ experience of association studies to understand the genetic architecture of pancreatic cancer. Crit Rev Oncol Hematol. 2023;186:104020. 37164172 10.1016/j.critrevonc.2023.104020

[94] Clarke TK, Adams MJ, Davies G, Howard DM, Hall LS, Padmanabhan S, et al. Genome-wide association study of alcohol consumption and genetic overlap with other health-related traits in UK Biobank (N=112 117). Mol Psychiatry. 2017;22(10):1376–84. 28937693 10.1038/mp.2017.153 PMC5622124

[95] Galimberti M, Levey DF, Deak JD, Zhou H, Stein MB, Gelernter J. Genetic influences and causal pathways shared between cannabis use disorder and other substance use traits. Mol Psychiatry. 2024;29(9):2905–10. 38580809 10.1038/s41380-024-02548-y PMC11419938

[96] Yang DW, Wang TM, Zhang JB, Li XZ, He YQ, Xiao R, et al. Genome-wide association study identifies genetic susceptibility loci and pathways of radiation-induced acute oral mucositis. J Transl Med. 2020;18(1):224. 32503578 10.1186/s12967-020-02390-0 PMC7275566

[97] Dong J, Maj C, Tsavachidis S, Ostrom QT, Gharahkhani P, Anderson LA, et al. Sex-Specific Genetic Associations for Barrett's Esophagus and Esophageal Adenocarcinoma. Gastroenterology. 2020;159(6):2065–76 e1. 10.1053/j.gastro.2020.08.052 PMC9057456 32918910

[98] Gerlinger M, Horswell S, Larkin J, Rowan AJ, Salm MP, Varela I, et al. Genomic architecture and evolution of clear cell renal cell carcinomas defined by multiregion sequencing. Nat Genet. 2014;46(3):225–33. 24487277 10.1038/ng.2891 PMC4636053

[99] Cuellar-Partida G, Lu Y, Dixon SC, Australian Ovarian Cancer S, Fasching PA, Hein A, et al. Assessing the genetic architecture of epithelial ovarian cancer histological subtypes. Hum Genet. 2016;135(7):741–56. 10.1007/s00439-016-1663-9 PMC4976079 27075448

[100] Ghouse J, Sveinbjornsson G, Vujkovic M, Seidelin AS, Gellert-Kristensen H, Ahlberg G, et al. Integrative common and rare variant analyses provide insights into the genetic architecture of liver cirrhosis. Nat Genet. 2024;56(5):827–37. 38632349 10.1038/s41588-024-01720-y PMC11096111

[101] Rosoff DB, Wagner J, Bell AS, Mavromatis LA, Jung J, Lohoff FW. A multi-omics Mendelian randomization study identifies new therapeutic targets for alcohol use disorder and problem drinking. Nat Hum Behav. 2024. 10.1038/s41562-024-02040-1. 39528761 10.1038/s41562-024-02040-1

[102] Toikumo S, Jennings MV, Pham BK, Lee H, Mallard TT, Bianchi SB, et al. Multi-ancestry meta-analysis of tobacco use disorder identifies 461 potential risk genes and reveals associations with multiple health outcomes. Nat Hum Behav. 2024;8(6):1177–93. 38632388 10.1038/s41562-024-01851-6 PMC11199106

[103] Matoba N, Akiyama M, Ishigaki K, Kanai M, Takahashi A, Momozawa Y, et al. GWAS of smoking behaviour in 165,436 Japanese people reveals seven new loci and shared genetic architecture. Nat Hum Behav. 2019;3(5):471–7. 31089300 10.1038/s41562-019-0557-y

[104] Friedman JR, Richbart SD, Merritt JC, Brown KC, Nolan NA, Akers AT, et al. Acetylcholine signaling system in progression of lung cancers. Pharmacol Ther. 2019;194:222–54. 30291908 10.1016/j.pharmthera.2018.10.002 PMC6348061

[105] Adams SJ, Stone E, Baldwin DR, Vliegenthart R, Lee P, Fintelmann FJ. Lung cancer screening. Lancet. 2023;401(10374):390–408. 36563698 10.1016/S0140-6736(22)01694-4

[106] Im PK, Wright N, Yang L, Chan KH, Chen Y, Guo Y, et al. Alcohol consumption and risks of more than 200 diseases in Chinese men. Nat Med. 2023;29(6):1476–86. 37291211 10.1038/s41591-023-02383-8 PMC10287564

[107] Jett J, Stone E, Warren G, Cummings KM. Cannabis use, lung cancer, and related issues. J Thorac Oncol. 2018;13(4):480–7. 29374567 10.1016/j.jtho.2017.12.013

[108] Zhang J, Peng S, Cheng H, Nomura Y, Di Narzo AF, Hao K. Genetic pleiotropy between nicotine dependence and respiratory outcomes. Sci Rep. 2017;7(1):16907. 29203782 10.1038/s41598-017-16964-4 PMC5715160

[109] Bray MJ, Chen LS, Fox L, Hancock DB, Culverhouse RC, Hartz SM, et al. Dissecting the genetic overlap of smoking behaviors, lung cancer, and chronic obstructive pulmonary disease: A focus on nicotinic receptors and nicotine metabolizing enzyme. Genet Epidemiol. 2020;44(7):748–58. 32803792 10.1002/gepi.22331 PMC7793026

[110] Beghini A, Magnani I, Roversi G, Piepoli T, Di Terlizzi S, Moroni RF, et al. The neural progenitor-restricted isoform of the MARK4 gene in 19q13.2 is upregulated in human gliomas and overexpressed in a subset of glioblastoma cell lines. Oncogene. 2003;22(17):2581–91. 12735302 10.1038/sj.onc.1206336

[111] Yin J, Rockenbauer E, Hedayati M, Jacobsen NR, Vogel U, Grossman L, et al. Multiple single nucleotide polymorphisms on human chromosome 19q13.2–3 associate with risk of Basal cell carcinoma. Cancer Epidemiol Biomarkers Prev. 2002;11(11):1449–53. 12433725

[112] Vogel U, Laros I, Jacobsen NR, Thomsen BL, Bak H, Olsen A, et al. Two regions in chromosome 19q13.2-3 are associated with risk of lung cancer. Mutat Res. 2004;546(1–2):65–74. 14757194 10.1016/j.mrfmmm.2003.11.001

[113] Yin J, Vogel U, Ma Y, Qi R, Wang H. Haplotypes of nine single nucleotide polymorphisms on chromosome 19q13.2-3 associated with susceptibility of lung cancer in a Chinese population. Mutat Res. 2008;641(1–2):12–8. 18358500 10.1016/j.mrfmmm.2008.02.004

[114] Berg M, Agesen TH, Thiis-Evensen E, group IN-s, Merok MA, Teixeira MR, et al. Distinct high resolution genome profiles of early onset and late onset colorectal cancer integrated with gene expression data identify candidate susceptibility loci. Mol Cancer. 2010;9:100. 10.1186/1476-4598-9-100 PMC2885343 20459617

[115] Kalthoff S, Ehmer U, Freiberg N, Manns MP, Strassburg CP. Coffee induces expression of glucuronosyltransferases by the aryl hydrocarbon receptor and Nrf2 in liver and stomach. Gastroenterology. 2010;139(5):1699–710, 710 e1-2. 20600030 10.1053/j.gastro.2010.06.048

[116] Josse AR, Da Costa LA, Campos H, El-Sohemy A. Associations between polymorphisms in the AHR and CYP1A1-CYP1A2 gene regions and habitual caffeine consumption. Am J Clin Nutr. 2012;96(3):665–71. 22854411 10.3945/ajcn.112.038794

[117] Gressner OA, Lahme B, Rehbein K, Siluschek M, Weiskirchen R, Gressner AM. Pharmacological application of caffeine inhibits TGF-beta-stimulated connective tissue growth factor expression in hepatocytes via PPARgamma and SMAD2/3-dependent pathways. J Hepatol. 2008;49(5):758–67. 18486259 10.1016/j.jhep.2008.03.029

[118] Rosendahl AH, Perks CM, Zeng L, Markkula A, Simonsson M, Rose C, et al. Caffeine and caffeic acid inhibit growth and modify estrogen receptor and insulin-like growth factor I receptor levels in human breast cancer. Clin Cancer Res. 2015;21(8):1877–87. 25691730 10.1158/1078-0432.CCR-14-1748

[119] Zarate LV, Pena Agudelo JA, Miret NV, Nicola Candia AJ, Perez Kupsilonper M, Pontillo CA, et al. Aryl hydrocarbon receptor-ligand hexachlorobenzene promotes immunosuppression leading to mammary tumor growth and metastasis in a HER2-positive syngeneic mouse model. Environ Toxicol Pharmacol. 2025;118:104796. 40834990 10.1016/j.etap.2025.104796

[120] Jiang X, Wang J, Lin L, Du L, Ding Y, Zheng F, et al. Macrophages promote pre-metastatic niche formation of breast cancer through aryl hydrocarbon receptor activity. Signal Transduct Target Ther. 2024;9(1):352. 39690159 10.1038/s41392-024-02042-5 PMC11652640

[121] Zhao B, Xin Z, Ren P, Wu H. The role of PPARs in breast cancer. Cells. 2022. 10.3390/cells12010130. 36611922 10.3390/cells12010130 PMC9818187

[122] Kim J, Munster PN. Estrogens and breast cancer. Ann Oncol. 2025;36(2):134–48. 39522613 10.1016/j.annonc.2024.10.824 PMC12168202

[123] Collaborators GBDCRF. The global burden of cancer attributable to risk factors, 2010-19: a systematic analysis for the Global Burden of Disease Study 2019. Lancet. 2022;400(10352):563–91. 35988567 10.1016/S0140-6736(22)01438-6 PMC9395583

[124] Colon-Echevarria CB, Lamboy-Caraballo R, Aquino-Acevedo AN, Armaiz-Pena GN. Neuroendocrine regulation of tumor-associated immune cells. Front Oncol. 2019;9:1077. 31737559 10.3389/fonc.2019.01077 PMC6828842

[125] Greten FR, Grivennikov SI. Inflammation and cancer: triggers, mechanisms, and consequences. Immunity. 2019;51(1):27–41. 31315034 10.1016/j.immuni.2019.06.025 PMC6831096

[126] Liu Y, Tian S, Ning B, Huang T, Li Y, Wei Y. Stress and cancer: the mechanisms of immune dysregulation and management. Front Immunol. 2022;13:1032294. 36275706 10.3389/fimmu.2022.1032294 PMC9579304

[127] Gruol DL. The neuroimmune system and the cerebellum. Cerebellum. 2023. 10.1007/s12311-023-01624-3. 37950146 10.1007/s12311-023-01624-3 PMC11585519

[128] Mandal SK, Yadav P, Sheth RA. The neuroimmune axis and its therapeutic potential for primary liver cancer. Int J Mol Sci. 2024. 10.3390/ijms25116237. 38892423 10.3390/ijms25116237 PMC11172507

[129] Chen F, Kang R, Tang D, Liu J. Ferroptosis: principles and significance in health and disease. J Hematol Oncol. 2024;17(1):41. 38844964 10.1186/s13045-024-01564-3 PMC11157757

[130] Poller WC, Downey J, Mooslechner AA, Khan N, Li L, Chan CT, et al. Brain motor and fear circuits regulate leukocytes during acute stress. Nature. 2022;607(7919):578–84. 35636458 10.1038/s41586-022-04890-z PMC9798885

[131] Xu FF, Huang Y, Wang XQ, Qiu YH, Peng YP. Modulation of immune function by glutamatergic neurons in the cerebellar interposed nucleus via hypothalamic and sympathetic pathways. Brain Behav Immun. 2014;38:263–71. 24583232 10.1016/j.bbi.2014.02.011

[132] Huang D, Alexander PB, Li QJ, Wang XF. Gabaergic signaling beyond synapses: an emerging target for cancer therapy. Trends Cell Biol. 2023;33(5):403–12. 36114091 10.1016/j.tcb.2022.08.004 PMC10008753

[133] Bellavance MA, Rivest S. The HPA - immune axis and the immunomodulatory actions of glucocorticoids in the brain. Front Immunol. 2014;5:136. 24744759 10.3389/fimmu.2014.00136 PMC3978367

[134] Kertser A, Baruch K, Deczkowska A, Weiner A, Croese T, Kenigsbuch M, et al. Corticosteroid signaling at the brain-immune interface impedes coping with severe psychological stress. Sci Adv. 2019;5(5):eaav4111. 31149632 10.1126/sciadv.aav4111 PMC6541460

[135] Aquino-Acevedo AN, Knochenhauer H, Castillo-Ocampo Y, Ortiz-Leon M, Rivera-Lopez YA, Morales-Lopez C, et al. Stress hormones are associated with inflammatory cytokines and attenuation of T-cell function in the ascites from patients with high grade serous ovarian cancer. Brain Behav Immun. 2022;26:100558. 10.1016/j.bbih.2022.100558 PMC9694096 36439058

[136] Curran L, Sharpe L, Butow P. Anxiety in the context of cancer: a systematic review and development of an integrated model. Clin Psychol Rev. 2017;56:40–54. 28686905 10.1016/j.cpr.2017.06.003

[137] Haywood D, Henry M, Dauer E, Lederman O, Farley M, Henneghan AM, et al. Cancer-related cognitive impairment as a key contributor to psychopathology in cancer survivors: implications for prevention, treatment and supportive care. Support Care Cancer. 2024;32(7):480. 38954104 10.1007/s00520-024-08696-9 PMC11219369

[138] Haywood D, O’Connor M, Baughman FD, Chan A, Chan RJ, Dauer E, et al. Protocol for the development and initial validation of the COG-IMPACT tool: a purpose-built unmet needs assessment for cancer-related cognitive impairment. Methods Protoc. 2024. 10.3390/mps7040054. 39051268 10.3390/mps7040054 PMC11270296

[139] Lotfipour S, Byun JS, Leach P, Fowler CD, Murphy NP, Kenny PJ, et al. Targeted deletion of the mouse alpha2 nicotinic acetylcholine receptor subunit gene (Chrna2) potentiates nicotine-modulated behaviors. J Neurosci. 2013;33(18):7728–41. 23637165 10.1523/JNEUROSCI.4731-12.2013 PMC3831006

[140] Lotfipour S, Mojica C, Nakauchi S, Lipovsek M, Silverstein S, Cushman J, et al. alpha2* Nicotinic acetylcholine receptors influence hippocampus-dependent learning and memory in adolescent mice. Learn Mem. 2017;24(6):231–44. 28507032 10.1101/lm.045369.117 PMC5435881

[141] Dash B, Lukas RJ, Li MD. A signal peptide missense mutation associated with nicotine dependence alters alpha2*-nicotinic acetylcholine receptor function. Neuropharmacology. 2014;79:715–25. 24467848 10.1016/j.neuropharm.2014.01.021 PMC3984001

[142] Demontis D, Rajagopal VM, Thorgeirsson TE, Als TD, Grove J, Leppala K, et al. Genome-wide association study implicates CHRNA2 in cannabis use disorder. Nat Neurosci. 2019;22(7):1066–74. 31209380 10.1038/s41593-019-0416-1 PMC7596896

[143] Paniagua-Herranz L, Moreno I, Nieto-Jimenez C, Garcia-Lorenzo E, Diaz-Tejeiro C, Sanvicente A, et al. Genomic and immunologic correlates in prostate cancer with high expression of KLK2. Int J Mol Sci. 2024. 10.3390/ijms25042222. 38396898 10.3390/ijms25042222 PMC10889228

[144] Goh JY, Rueda P, Taylor J, Rathbone A, Scott D, Langmead CJ, et al. Transcriptomic analysis of rat prefrontal cortex following chronic stress induced by social isolation - Relevance to psychiatric and neurodevelopmental illness, and implications for treatment. Neurobiol Stress. 2024;33:100679. 39502833 10.1016/j.ynstr.2024.100679 PMC11536066

[145] She X, Gao Y, Zhao Y, Yin Y, Dong Z. A high-throughput screen identifies inhibitors of lung cancer stem cells. Biomed Pharmacother. 2021;140:111748. 34044271 10.1016/j.biopha.2021.111748

[146] Panula P. Histamine, histamine H(3) receptor, and alcohol use disorder. Br J Pharmacol. 2020;177(3):634–41. 30801695 10.1111/bph.14634 PMC7012945

[147] Krementsov DN, Wall EH, Martin RA, Subramanian M, Noubade R, Del Rio R, et al. Histamine H(3) receptor integrates peripheral inflammatory signals in the neurogenic control of immune responses and autoimmune disease susceptibility. PLoS One. 2013;8(7):e62743. 23894272 10.1371/journal.pone.0062743 PMC3718788

[148] Lin L, Gong S, Deng C, Zhang G, Wu J. PTK6: an emerging biomarker for prognosis and immunotherapeutic response in clear cell renal carcinoma (KIRC). Heliyon. 2024;10(7):e29001. 38596018 10.1016/j.heliyon.2024.e29001 PMC11002233

[149] Ito K, Park SH, Katsyv I, Zhang W, De Angelis C, Schiff R, et al. PTK6 regulates growth and survival of endocrine therapy-resistant ER+ breast cancer cells. NPJ Breast Cancer. 2017;3:45. 29167821 10.1038/s41523-017-0047-1 PMC5694002

[150] Lang YD, Chen HY, Ho CM, Shih JH, Hsu EC, Shen R, et al. PSPC1-interchanged interactions with PTK6 and beta-catenin synergize oncogenic subcellular translocations and tumor progression. Nat Commun. 2019;10(1):5716. 31844057 10.1038/s41467-019-13665-6 PMC6914800

[151] Ito K, Park SH, Nayak A, Byerly JH, Irie HY. PTK6 inhibition suppresses metastases of triple-negative breast cancer via SNAIL-dependent E-cadherin regulation. Cancer Res. 2016;76(15):4406–17. 27302163 10.1158/0008-5472.CAN-15-3445

[152] Regan Anderson TM, Ma SH, Raj GV, Cidlowski JA, Helle TM, Knutson TP, et al. Breast Tumor Kinase (Brk/PTK6) Is Induced by HIF, Glucocorticoid Receptor, and PELP1-Mediated Stress Signaling in Triple-Negative Breast Cancer. Cancer Res. 2016;76(6):1653–63. 26825173 10.1158/0008-5472.CAN-15-2510 PMC4794366

[153] Wozniak DJ, Kajdacsy-Balla A, Macias V, Ball-Kell S, Zenner ML, Bie W, et al. PTEN is a protein phosphatase that targets active PTK6 and inhibits PTK6 oncogenic signaling in prostate cancer. Nat Commun. 2017;8(1):1508. 29142193 10.1038/s41467-017-01574-5 PMC5688148

[154] Gilic MB, Tyner AL. Targeting protein tyrosine kinase 6 in cancer. Biochim Biophys Acta Rev Cancer. 2020;1874(2):188432. 32956764 10.1016/j.bbcan.2020.188432 PMC7716696

[155] Shi X, Li M, Yao J, Li MD, Yang Z. Alcohol drinking, DNA methylation and psychiatric disorders: a multi-omics Mendelian randomization study to investigate causal pathways. Addiction. 2024;119(7):1226–37. 38523595 10.1111/add.16465

[156] Ochi R, Ueno F, Sakuma M, Tani H, Tsugawa S, Graff-Guerrero A, et al. Patterns of functional connectivity alterations induced by alcohol reflect somatostatin interneuron expression in the human cerebral cortex. Sci Rep. 2022;12(1):7896. 35550587 10.1038/s41598-022-12035-5 PMC9098480

[157] Zhou JL, de Guglielmo G, Ho AJ, Kallupi M, Pokhrel N, Li HR, et al. Single-nucleus genomics in outbred rats with divergent cocaine addiction-like behaviors reveals changes in amygdala GABAergic inhibition. Nat Neurosci. 2023;26(11):1868–79. 37798411 10.1038/s41593-023-01452-y PMC10620093

[158] Barr J, Walz A, Restaino AC, Amit M, Barclay SM, Vichaya EG, et al. Tumor-infiltrating nerves functionally alter brain circuits and modulate behavior in a mouse model of head-and-neck cancer. Elife. 2024. 10.7554/eLife.97916.3. 39302290 10.7554/eLife.97916 PMC11415076

[159] Peterson RE, Kuchenbaecker K, Walters RK, Chen CY, Popejoy AB, Periyasamy S, et al. Genome-wide association studies in ancestrally diverse populations: opportunities, methods, pitfalls, and recommendations. Cell. 2019;179(3):589–603. 31607513 10.1016/j.cell.2019.08.051 PMC6939869

[160] Corpas M, Pius M, Poburennaya M, Guio H, Dwek M, Nagaraj S, et al. Bridging genomics’ greatest challenge: the diversity gap. Cell Genom. 2025;5(1):100724. 39694036 10.1016/j.xgen.2024.100724 PMC11770215

