# Supplementary material for: Shared genetic architecture of psychoactive substance use and pan-cancer: insights from a large‑scale genome‑wide cross‑trait analysis
Source: BMC Med. 2026 Feb 5;24:124. doi: 10.1186/s12916-026-04677-3 (PMC12930682; doi:10.1186/s12916-026-04677-3)
Supplement: Supplementary file 1 — Additional file 1. Figures S1–S3. [file 12916_2026_4677_MOESM1_ESM.docx]

Additional file 1

Supplementary Figures for:

**Shared Genetic Architecture of Psychoactive Substance Use and Pan-Cancer: Insights from a Large‑Scale Genome‑Wide Cross‑Trait Analysis**

Jiahang Song^1#^, Pengzhu Li^2#^, Martin Canis^1^, Kristian Unger^3^, Nikolaus Alexander Haas^2^, Olivier Gires^1^

**Fig. S1**. Circular networks showing the pleiotropic landscape between (A) nicotine use dependence (NicUD), (B) cannabis use disorder (CanUD), (C) opioid use (OpiU), (D) coffee use (CofU), and (E) tea use (TeaU) and cancer.

Abbreviations: bladder carcinoma (BLCA), lung cancer (LC), lung adenocarcinoma (LUAD), cervival squamous cell carcinoma (CESC), lung squamous cell carcinoma (LUSC), estrogen receptor-negative breast cancer (BRCA-ER-), breast cancer (BRCA), estrogen receptor-positive breast cancer (BRCA-ER+), esophageal adenocarcinoma (EAC). The circular network includes SNP-affected chromosomal loci (yellow circles) and genes (blue circles) and distinguishes shared and causal loci (open/closed circles), and shared genes identified by positional mapping and by MAGMA analysis (open/closed circles).

**Fig. S2**. Gene set enrichment from the MAGMA analysis of pleiotropic loci. Shown are gene sets enriched with respect to pleiotropic loci associated with (A) alcohol use dependence (AlcUD), (B) nicotine use dependence (NicUD), (C) cannabis use disorder (CanUD), (D) opioid us (OpiU), coffee use (CofU), and (E) tea use (TeaU) with cancer according to significance.

Abbreviations: bladder carcinoma (BLCA), lung cancer (LC), lung adenocarcinoma (LUAD), cervical squamous cell carcinoma (CESC), lung squamous cell carcinoma (LUSC), estrogen receptor-negative breast cancer (BRCA-ER-), breast cancer (BRCA), estrogen receptor-positive breast cancer (BRCA-ER+), esophageal adenocarcinoma (EAC), ovarian adenocarcinoma (OCAC).

**Fig. S3**. UMAP representation of single-cell disease relevance z-scores (scDRS) of psychoactive substances and cancer trait-pairs including alcohol use dependence (AlcUD; A), nicotine use dependence (NicUD; B), cannabis use disorder (CanUD, C), opioid use (OpiU; D), and tea use (TeaU; E). Color coding represents the disease score, where a darker color indicates a higher score, signifying greater disease enrichment for the specific cell cluster. Red: enrichment; blue: repression.
