# Supplementary material for: Shared genetic architecture of psychoactive substance use and pan-cancer: insights from a large‑scale genome‑wide cross‑trait analysis
Source: BMC Med. 2026 Feb 5;24:124. doi: 10.1186/s12916-026-04677-3 (PMC12930682; doi:10.1186/s12916-026-04677-3)
Supplement: Supplementary file 3 — Additional file 3. STROBE-MR-checklist. [file 12916_2026_4677_MOESM3_ESM.docx]

**STROBE-MR checklist of recommended items to address in reports of Mendelian randomization studies**^1^ ^2^

| **Item No.** | **Section** | **Checklist item** | **Page No.** | **Relevant text from manuscript** |
| --- | --- | --- | --- | --- |
| 1 | **TITLE and ABSTRACT** | Indicate Mendelian randomization (MR) as the study’s design in the title and/or the abstract if that is a main purpose of the study | 2 | Mendelian randomization (MR) analysis was employed to explore the causal associations between PSU and cancer.  MR analysis identified causal effects of PSU (AlcUD and NicUD) on cancer risk. |
|  | **INTRODUCTION** |  |  |  |
| 2 | **Background** | Explain the scientific background and rationale for the reported study. What is the exposure? Is a potential causal relationship between exposure and outcome plausible? Justify why MR is a helpful method to address the study question | 3 | Substance use disorders (SUD) and psychoactive substance use (PSU) are very serious conditions that are associated with high relapse rates and morbidity. SUD/PSU have an additional health impact as they potentiate the development of illnesses including chronic pain, cardiovascular diseases, mental health disorders, and cancer [1-6]. The term cancer covers over 200 malignant diseases with pronounced inter- and intratumor molecular heterogeneity and variations in treatment response due to diverse underlying pathobiological, molecular mechanisms, and co-morbidities. PSU and tumorigenesis are both driven to varying degrees by genetic, epigenetic, environmental, and behavioral factors, and together cause major health and economic problems worldwide [1, 4, 7, 8]. |
| 3 | **Objectives** | State specific objectives clearly, including pre-specified causal hypotheses (if any). State that MR is a method that, under specific assumptions, intends to estimate causal effects | 4-5 | Traditional observational studies are inherently limited by residual confounding, selection bias, and measurement errors, which may obscure true relationships between PSU and cancer risk. MR methods uncovered causal effects of PSU (AlcUD and NicUD) on cancer risk. |
|  | **METHODS** |  |  |  |
| 4 | **Study design and data sources** | Present key elements of the study design early in the article. Consider including a table listing sources of data for all phases of the study. For each data source contributing to the analysis, describe the following: |  |  |
|  | a) | Setting: Describe the study design and the underlying population, if possible. Describe the setting, locations, and relevant dates, including periods of recruitment, exposure, follow-up, and data collection, when available. | Fig.6A  Table S1 |  |
|  | b) | Participants: Give the eligibility criteria, and the sources and methods of selection of participants. Report the sample size, and whether any power or sample size calculations were carried out prior to the main analysis | 5 | (i) GWAS summary statistics for alcohol use dependence (AlcUD) and cannabis use disorder (CanUD) were obtained from the Psychiatric Genomics Consortium (PGC). Summary statistics for nicotine use dependence (NicUD) were collected from the GWAS and Sequencing Consortium of Alcohol and Nicotine Use (GSCAN). Summary statistics for aspirin use (AspU), opioid use (OpiU), coffee use (CofU), and tea use (TeaU) were obtained from the UK Biobank. Summary statistics for hypnotics use (HypU) were collected from the FinnGen R11 database.  (ii) GWAS summary statistics for 20 types of cancer were retrieved from public large-scale GWAS or GWAS meta-analyses: glioma (GLIOMA), low-grade gliomas (LGG), glioblastoma (GBM), esophageal adenocarcinoma (EAC), lung cancer (LC) , lung adenocarcinoma (LUAD), lung squamous cell carcinoma (LUSC), small cell lung cancer (SCLC), breast cancer (BRCA), breast cancer with estrogen receptor-positive status (BRCA ER+), breast cancer with estrogen receptor-negative status (BRCA ER-), colon cancer (COAD), rectum cancer (READ), bladder cancer (BLCA), renal cell carcinoma (RCC), cervical cancer (CESC), uterine corpus endometrial cancer (UCEC), malignant melanoma (MELA), prostate cancer (PRAD), and ovarian cancer (OCAC). |
|  | c) | Describe measurement, quality control and selection of genetic variants | 6 | All GWAS summary statistics were harmonized to the GRCh37 (hg19) reference genome prior to analysis. SNP-level quality control was performed following standard GWAS practices to ensure data consistency across traits. Filtering criteria are: (i) exclusion of non-bipartite allele SNPs and SNPs with strand-ambiguous alleles; (ii) exclusion of SNPs without rs tags; (iii) deletion of duplicated SNPs, SNPs excluded in the 1,000 Genomes Project, or with mismatched alleles; (iv) exclusion of SNPs within major histocompatibility complex region at chr6: 28.5–33.5Mb from LDSC analysis; (v) retention of SNPs with minor allele frequency (MAF) > 0.01. |
|  | d) | For each exposure, outcome, and other relevant variables, describe methods of assessment and diagnostic criteria for diseases | 5 | (i) GWAS summary statistics for alcohol use dependence (AlcUD) and cannabis use disorder (CanUD) were obtained from the Psychiatric Genomics Consortium (PGC). Summary statistics for nicotine use dependence (NicUD) were collected from the GWAS and Sequencing Consortium of Alcohol and Nicotine Use (GSCAN). Summary statistics for aspirin use (AspU), opioid use (OpiU), coffee use (CofU), and tea use (TeaU) were obtained from the UK Biobank. Summary statistics for hypnotics use (HypU) were collected from the FinnGen R11 database.  (ii) GWAS summary statistics for 20 types of cancer were retrieved from public large-scale GWAS or GWAS meta-analyses: glioma (GLIOMA), low-grade gliomas (LGG), glioblastoma (GBM), esophageal adenocarcinoma (EAC), lung cancer (LC) , lung adenocarcinoma (LUAD), lung squamous cell carcinoma (LUSC), small cell lung cancer (SCLC), breast cancer (BRCA), breast cancer with positive estrogen receptor (BRCA ER+), breast cancer with negative estrogen receptor (BRCA ER-), colon cancer (COAD), rectum cancer (READ), bladder cancer (BLCA), renal cell carcinoma (RCC), cervical cancer (CESC), uterine corpus endometrial cancer (UCEC), malignant melanoma (MELA), prostate cancer (PRAD), and ovarian cancer (OCAC). |
|  | e) | Provide details of ethics committee approval and participant informed consent, if relevant | 23 | **Ethics statement** |
| 5 | **Assumptions** | Explicitly state the three core IV assumptions for the main analysis (relevance, independence and exclusion restriction) as well assumptions for any additional or sensitivity analysis | Fig.6A |  |
| 6 | **Statistical methods: main analysis** | Describe statistical methods and statistics used |  |  |
|  | a) | Describe how quantitative variables were handled in the analyses (i.e., scale, units, model) | 10 | Causal effects between each trait-pair were assessed using four MR methods: inverse variance weighted (IVW) [66], MR Egger [67], RAPS [68], and CAUSE [69]. Cochran’s Q statistics were applied to detect the effect size heterogeneity across IVs |
|  | b) | Describe how genetic variants were handled in the analyses and, if applicable, how their weights were selected | 10 | Two-sample Mendelian randomization (MR) analysis was conducted to determine potential causal effects between PSU and cancer traits. Linkage disequilibrium (r²) clumping was employed in PLINK 1.9 to obtain independent significance SNPs (P < 5 × 10⁻⁸) for all exposure traits, using an r² threshold of 0.001 within a 10,000 kb window. |
|  | c) | Describe the MR estimator (e.g. two-stage least squares, Wald ratio) and related statistics. Detail the included covariates and, in case of two-sample MR, whether the same covariate set was used for adjustment in the two samples | 10 | For each genetic instrument, causal effects were estimated using the Wald ratio and combined across variants using the IVW method. MR-Egger regression was applied allowing for a non-zero intercept to account for directional pleiotropy. RAPS was used to obtain robust estimates in the presence of weak instruments, and CAUSE was implemented as a Bayesian approach modeling both correlated and uncorrelated pleiotropic effects. |
|  | d) | Explain how missing data were addressed |  | NA |
|  | e) | If applicable, indicate how multiple testing was addressed | 10 | A false discovery rate (FDR) of 5% was used as the threshold. |
| 7 | **Assessment of assumptions** | Describe any methods or prior knowledge used to assess the assumptions or justify their validity | 10 | The strength of the instrumental variables was evaluated using the proportion of variance explained (PVE) and the F statistic (F > 10) |
| 8 | **Sensitivity analyses and additional analyses** | Describe any sensitivity analyses or additional analyses performed (e.g. comparison of effect estimates from different approaches, independent replication, bias analytic techniques, validation of instruments, simulations) | 10 | Causal effects between each trait-pair were assessed using four MR methods: inverse variance weighted (IVW) [66], MR Egger [67], RAPS [68], and CAUSE [69]. Cochran’s Q statistics were applied to detect the effect size heterogeneity across the IVs [70]. |
| 9 | **Software and pre-registration** |  |  |  |
|  | a) | Name statistical software and package(s), including version and settings used | 11 | Two-sample MR analysis was conducted with “MendelianRandomization” (version v0.10.0) [76]. |
|  | b) | State whether the study protocol and details were pre-registered (as well as when and where) |  | NA |
|  | **RESULTS** |  |  |  |
| 10 | **Descriptive data** |  |  |  |
|  | a) | Report the numbers of individuals at each stage of included studies and reasons for exclusion. Consider use of a flow diagram | Table S1 |  |
|  | b) | Report summary statistics for phenotypic exposure(s), outcome(s), and other relevant variables (e.g. means, SDs, proportions) | Fig.6A |  |
|  | c) | If the data sources include meta-analyses of previous studies, provide the assessments of heterogeneity across these studies |  | NA |
|  | d) | For two-sample MR:  i.  Provide justification of the similarity of the genetic variant-exposure associations between the exposure and outcome samples  ii.  Provide information on the number of individuals who overlap between the exposure and outcome studies | Table S1 | All study populations listed in Supplementary Table 1 were of European ancestry, resulting in minimal population heterogeneity with respect to ancestry. |
| 11 | **Main results** |  |  |  |
|  | a) | Report the associations between genetic variant and exposure, and between genetic variant and outcome, preferably on an interpretable scale | 15  Table S9  Table S10 | The risk of OCAC was observed to increase with higher genetic liability to AlcUD, with the causal effect assessed by the IVW method (OR = 1.216, 95%CI = 1.082–1.366, P = 0.001). Additionally, MR revealed a causal effect of NicUD on LC risk (IVW, OR = 1.124, 95%CI = 1.010–1.251, P = 0.032).  Table S9. Causal association analysis by MR method. Table S10. Summary of CAUSE results. |
|  | b) | Report MR estimates of the relationship between exposure and outcome, and the measures of uncertainty from the MR analysis, on an interpretable scale, such as odds ratio or relative risk per SD difference | 15  Table S9  Table S10 | The risk of OCAC was observed to increase with higher genetic liability to AlcUD, with the causal effect assessed by the IVW method (OR = 1.216, 95%CI = 1.082–1.366, P = 0.001). Additionally, MR revealed a causal effect of NicUD on LC risk (IVW, OR = 1.124, 95%CI = 1.010–1.251, P = 0.032).  Table S9. Causal association analysis by MR method. Table S10. Summary of CAUSE results. |
|  | c) | If relevant, consider translating estimates of relative risk into absolute risk for a meaningful time period |  | NA |
|  | d) | Consider plots to visualize results (e.g. forest plot, scatterplot of associations between genetic variants and outcome versus between genetic variants and exposure) | Fig. 6 |  |
| 12 | **Assessment of assumptions** |  |  |  |
|  | a) | Report the assessment of the validity of the assumptions | Table S9 | Table S9. Causal association analysis by MR method. |
|  | b) | Report any additional statistics (e.g., assessments of heterogeneity across genetic variants, such as *I^2^*, Q statistic or E-value) | Table S9 | Table S9. Causal association analysis by MR method. |
| 13 | **Sensitivity analyses and additional analyses** |  |  |  |
|  | a) | Report any sensitivity analyses to assess the robustness of the main results to violations of the assumptions | Table S9 | Table S9. Causal association analysis by MR method. |
|  | b) | Report results from other sensitivity analyses or additional analyses | Table S9 | Table S9. Causal association analysis by MR method. |
|  | c) | Report any assessment of direction of causal relationship (e.g., bidirectional MR) | Table S9 | Table S9. Causal association analysis by MR method. |
|  | d) | When relevant, report and compare with estimates from non-MR analyses |  | NA |
|  | e) | Consider additional plots to visualize results (e.g., leave-one-out analyses) |  | NA |
|  | **DISCUSSION** |  |  |  |
| 14 | **Key results** | Summarize key results with reference to study objectives | 18 | Our MR analysis further supports a causal relationship between PSU and cancer risk. Specifically, results based on the IVW method showed that increased genetic liability to AlcUD was significantly associated with a higher risk of OCAC, while NicUD exhibited a significant causal effect on LC |
| 15 | **Limitations** | Discuss limitations of the study, taking into account the validity of the IV assumptions, other sources of potential bias, and imprecision. Discuss both direction and magnitude of any potential bias and any efforts to address them | 20 | The genetic architecture of illnesses and their linkage depend on genetic ancestry, which is not covered in an unbiased manner in current GWAS, as studies include mostly individuals of European ancestry [82, 149, 150]. Further, a medical benefit of targeting hub genes associated with PSU-cancer trait-pairs remains to be validated in experimental and clinical settings, which represents a challenging task. |
| 16 | **Interpretation** |  |  |  |
|  | a) | Meaning: Give a cautious overall interpretation of results in the context of their limitations and in comparison with other studies | 18 | The causal links identified in this study highlight the importance of PSU as a modifiable behavioral risk factor in cancer prevention. |
|  | b) | Mechanism: Discuss underlying biological mechanisms that could drive a potential causal relationship between the investigated exposure and the outcome, and whether the gene-environment equivalence assumption is reasonable. Use causal language carefully, clarifying that IV estimates may provide causal effects only under certain assumptions | 18 | Coffee and its bioactive components, such as caffeine, have been shown to regulate multiple nuclear receptors, including the aryl hydrocarbon receptor (AHR) [105, 106], peroxisome proliferator-activated receptors (PPARs) [107], and estrogen receptor (ER) signaling [108]. These receptors are involved in caffeine metabolism and are also closely associated with the development and progression of breast cancer [109-112]. Therefore, genetic variations that affect coffee metabolism or response may influence breast cancer susceptibility by modulating nuclear receptor-mediated transcriptional networks. |
|  | c) | Clinical relevance: Discuss whether the results have clinical or public policy relevance, and to what extent they inform effect sizes of possible interventions | 20 | Thus, our findings suggest an implication of the PSU-cancer genetic architecture in influencing behavioral and non-behavioral aspects with repercussions on substance use and cancer [146-148]. Inhibition of hub genes with pleiotropic effects in PSU-cancer trait-pairs as adjuvant co-treatment may help prevent comorbidities entailed by trait linkage. This is particularly of interest with respect to cannabinoids and opioids, as they are in use for the treatment of cancer patients to enhance appetite and reduce disease-entailed pain. Furthermore, defined SNPs and hub gene expressions have potential to identify patients suffering from psychoactive drug abuse or from cancer who are at increased risk to develop respectively linked illnesses. Such stratification may provide a framework for improved personalized medicine and early detection to improve the prognosis of patients. |
| 17 | **Generalizability** | Discuss the generalizability of the study results (a) to other populations, (b) across other exposure periods/timings, and (c) across other levels of exposure | 20 | The genetic architecture of illnesses and their linkage depend on genetic ancestry, which is not covered in an unbiased manner in current GWAS, as studies include mostly individuals of European ancestry [82, 149, 150]. Future studies are needed in non-European populations. |
|  | **OTHER INFORMATION** |  |  |  |
| 18 | **Funding** | Describe sources of funding and the role of funders in the present study and, if applicable, sources of funding for the databases and original study or studies on which the present study is based | 23 | JS and PL received scholarships from the Chinese Scholarship Council. |
| 19 | **Data and data sharing** | Provide the data used to perform all analyses or report where and how the data can be accessed, and reference these sources in the article. Provide the statistical code needed to reproduce the results in the article, or report whether the code is publicly accessible and if so, where | 23 | Data Availability |
| 20 | **Conflicts of Interest** | All authors should declare all potential conflicts of interest | 23 | The authors have no conflict of interest to declare. |

This checklist is copyrighted by the Equator Network under the Creative Commons Attribution 3.0 Unported (CC BY 3.0) license.

1. Skrivankova VW, Richmond RC, Woolf BAR, Yarmolinsky J, Davies NM, Swanson SA, et al. Strengthening the Reporting of Observational Studies in Epidemiology using Mendelian Randomization (STROBE-MR) Statement. JAMA. 2021;under review.

2. Skrivankova VW, Richmond RC, Woolf BAR, Davies NM, Swanson SA, VanderWeele TJ, et al. Strengthening the Reporting of Observational Studies in Epidemiology using Mendelian Randomisation (STROBE-MR): Explanation and Elaboration. BMJ. 2021;375:n2233.
